# Criteria for central respiratory chemoreceptors: experimental evidence supporting current candidate cell groups

**DOI:** 10.3389/fphys.2023.1241662

**Published:** 2023-09-01

**Authors:** Elizabeth C. Gonye, Douglas A. Bayliss

**Affiliations:** Department of Pharmacology, University of Virginia, Charlottesville, VA, United States

**Keywords:** central chemosensitivity, hypercapnic ventilatory response, interoception, respiratory control, chemoreceptor

## Abstract

An interoceptive homeostatic system monitors levels of CO_2_/H^+^ and provides a proportionate drive to respiratory control networks that adjust lung ventilation to maintain physiologically appropriate levels of CO_2_ and rapidly regulate tissue acid-base balance. It has long been suspected that the sensory cells responsible for the major CNS contribution to this so-called respiratory CO_2_/H^+^ chemoreception are located in the brainstem—but there is still substantial debate in the field as to which specific cells subserve the sensory function. Indeed, at the present time, several cell types have been championed as potential respiratory chemoreceptors, including neurons and astrocytes. In this review, we advance a set of criteria that are necessary and sufficient for definitive acceptance of any cell type as a respiratory chemoreceptor. We examine the extant evidence supporting consideration of the different putative chemoreceptor candidate cell types in the context of these criteria and also note for each where the criteria have not yet been fulfilled. By enumerating these specific criteria we hope to provide a useful heuristic that can be employed both to evaluate the various existing respiratory chemoreceptor candidates, and also to focus effort on specific experimental tests that can satisfy the remaining requirements for definitive acceptance.

## 1 Introduction

The respiratory control system is responsible for homeostatic regulation of blood gases and rapid control of tissue pH, with dedicated sensors to detect the principal regulated variables, O_2_ and CO_2_/H^+^, and drive the appropriate ventilatory responses. It has long been known that O_2_ sensing is mediated primarily by the carotid bodies, with Corneille Heymans winning the Nobel Prize in 1938 for this discovery; the molecular mechanisms by which carotid glomus cells sense hypoxia remains an area of active investigation (Buckler, 2015; Mokashi et al., 2021; López-Barneo, 2022). It has also been long known that detection of CO_2_/H^+^ takes place mainly in the brainstem. However, in this case the cellular identity of the relevant chemosensors has remained elusive, and thus the cellular and molecular mechanisms for CO_2_/H^+^ detection have been less clear.

The hunt for central chemoreceptors has been active for more than a century, at least since the description of the hypercapnic ventilatory reflex (HCVR) by Haldane and Priestly in 1905 (Haldane and Priestley, 1905). Subsequent research pointed to the brainstem as the most likely site for the cells controlling the chemoreflex, and various inventive approaches have been used to examine CO_2_/H^+^ sensitivity in various brainstem regions and link the putatively chemosensitive cells in those regions to breathing regulation. Historically, these approaches have included: determining *in vivo* activation of cells by CO_2_, often via proxy measures such as Fos expression; identifying CO_2_/H^+^ sensitive cells, mostly using various *in vitro* preparations; measuring effects of focal acidification on breathing *in vivo*; and examining effects of localized, but relatively non-specific, chemotoxic lesions on respiration and the HCVR (Feldman et al., 2003). Informed by these approaches, some initial evidentiary criteria were enumerated for establishing respiratory chemoreceptor sites, and this yielded support for multiple regions/cell types to be proposed as respiratory chemoreceptors, with each contributing differentially under specific physiological conditions (e.g., during sleep and wake) (Feldman et al., 2003). At the same time, however, appropriate cautions regarding the criteria engendered by those experimental approaches, with their inherent limitations, were already apparent (Feldman et al., 2003). Since then, there have been staggering technological advances that have allowed precise phenotypic characterization and genetic access to distinct cell types, cell-specific manipulation of activity using novel optogenetic and chemogenetic tools, and molecular identification of putative substrates for CO_2_/H^+^ detectors. These new approaches obviate some of the earlier limitations and also permit elaboration of a more exacting set of criteria for defining a cell as a respiratory chemoreceptor.

We have proposed the following set of five criteria that can be used to standardize the interpretation of work to identify central chemoreceptors (Guyenet and Bayliss, 2022): 1) activation and inhibition of the candidate cell group have opposite effects on respiration; 2) inhibition of candidate cells blunts the respiratory response to CO_2_; 3) cell activity *in vivo* tracks pH or PCO_2_; 4) CO_2_/H^+^ modulation of cell activity is a direct effect, at least in part; and 5) interfering with the specific molecular mechanism(s) by which a cell senses CO_2_/H^+^ inhibits the normal hypercapnic ventilatory response (Guyenet and Bayliss, 2022). Conditions 1 and 2 are obviously necessary, but also not sufficient—i.e., it is possible to obtain those effects by acting on component(s) of the respiratory system downstream of the actual “chemoreceptors.” Similarly, condition 3 is also necessary but not sufficient since alterations in cell activity could be solely due to synaptic mechanisms and not reflect an intrinsic CO_2_/H^+^ sensitivity that would be required for a true “sensor.” Conditions 4 and 5 are the most stringent and address the specific molecular mechanisms by which cells sense and respond to CO_2_/H^+^. Condition 5 is the only criterion that is both necessary and sufficient. This review will focus on the primary cell groups that have been put forward as chemoreceptors, evaluate whether the existing evidence satisfies these new criteria, and identify gaps in knowledge that remain to be filled.

## 2 Retrotrapezoid nucleus

**FIGURE 1:**
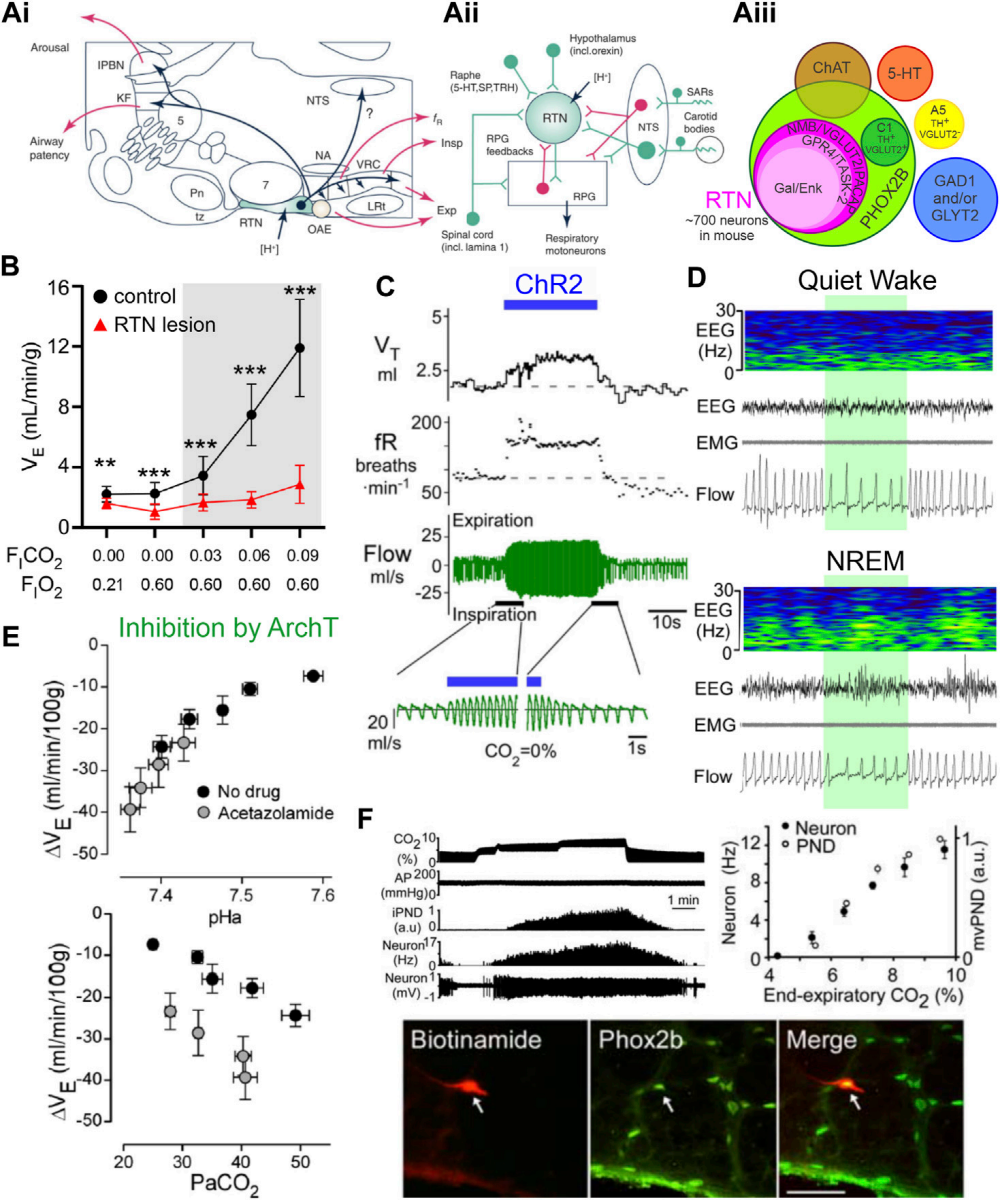
Phox2b-expressing retrotrapezoid nucleus neurons are activated by CO_2_/H^+^, drive respiration, and are necessary for CO_2_ stimulated breathing. **(A)(i)** Sagittal brainstem schematic showing location of the RTN (green) with key projections highlighted in blue; abbreviations: lateral parabrachial nuclei (lPBN), Kölliker-Fuse (KF), nucleus of the solitary tract (NTS), trigeminal motor nucleus (5) nucleus ambiguus (NA), ventral respiratory column (VRC) containing from rostral to caudal the Bötzinger, preBötzinger (preBötC), and the rostral and caudal divisions of the ventral respiratory group (rVRG/cVRG), pontine nuclei (Pn), facial motor nucleus (7) trapezoid body (tz), retrotrapezoid nucleus (RTN), oscillator for active expiration (OAE), lateral reticular nucleus (LRt). **(A)(ii)** Model of RTN highlighting different respiratory related inputs and output to the respiratory pattern generator (RPG, including the preBötC), abbreviations: 5-hydroxytryptophan (5-HT, serotonin), substance P (SP), thyrotrophin releasing hormone (TRH), stretch activated receptors (SARs). **(A)(iii)** Current molecular definition of RTN neurons; abbreviations: choline acetyltransferase (ChAT), glutamate dehydroxylase (GAD), glycine transporter 2 (GLYT2), tyrosine hydroxylase (TH), Neuromedin B (NMB), vesicular glutamate transporter (VGLUT2), pituitary adenylate cyclase activating peptide (PACAP), G-protein coupled receptor 4 (GPR4), TWIK-related acid sensitive channel 2 (TASK-2), galanin (Gal), enkephalin (Enk). Panels i and ii adapted from Guyenet et al. (2019), Figure 2; panel **(iii)** adapted from (Shi et al., 2017), Figure 12. **(B)** Near complete lesion of RTN neurons using Cre-dependent caspase expression in NMB-Cre mice nearly eliminates the HCVR in conscious mice. Panel adapted from (Souza et al., 2023), Figure 5D. **(C)** Acute activation of Phox2b-expressing RTN neurons by ChR2 stimulation (blue bar) in conscious rats drives respiration via increases in tidal volume and frequency. Panel adapted from (Abbott et al., 2011), Figure 1B. **(D)** Acute inhibition of NMB-expressing RTN neurons via activation of the inhibitory opsin ArchT (shaded green) decreases respiration during wake and non-REM sleep. Panel adapted from (Souza et al., 2023), Figure 7D. **(E)** Inhibition of Phox2b-expressing RTN neurons with the inhibitory opsin ArchT reduces V_E_; this effect depends on arterial pH (upper) or PaCO_2_ (lower) and tracks most closely with pHa. The more pronounced inhibition at lower pHa reflects greater RTN neuron activity. Panel adapted from (Basting et al., 2015), Figures 11C, F. **(F)** Phox2b-expressing RTN neuron activity tracks inspired CO_2_ level when measured by extracellular recording in anesthetized rats *in vivo*; example of recorded RTN neuron (biotinamide) that was immunopositive for Phox2b. Panel adapted from (Stornetta et al., 2006), Figures 2A, C, D, copyright 2006 Society for Neuroscience.

The retrotrapezoid nucleus (RTN) was first identified as a group of cells near the ventral surface of the rostral medulla, inferior to the facial motor nucleus and posterior to the trapezoid bodies, that project to the dorsal respiratory group (DRG) and ventral respiratory group (VRG) in the brainstem (Smith et al., 1989; Connelly et al., 1990; Ellenberger and Feldman, 1990). The anatomical location of these RTN neurons coincided well with an acid-sensitive region of the rostral ventral medullary surface first identified in 1963 (Mitchell et al., 1963), prompting an early and prescient speculation that RTN neurons might be the relevant anatomical substrate for these respiratory chemoreceptors (Smith et al., 1989). It is now known that RTN neurons project to various respiratory-related regions, including the preBötzinger complex (preBötC), nucleus of the solitary tract (NTS), Kolliker Fuse (KF), and the lateral parabrachial nucleus (lPBN). This region also receives diverse chemical inputs from the NTS, the medullary and dorsal raphe nuclei, KF, A5, and the lPBN (Figures 1Ai-Aii) (Rosin et al., 2006; Bochorishvili et al., 2012).

As mentioned, the RTN appellation was originally applied to cells in the parafacial region that project to the DRG and VRG. The RTN name has been used by some groups to reference the parafacial region more generally, including all the various cells located therein. We choose a more restrictive definition, to respect both the initial hodological definition of RTN neurons and to acknowledge the subsequent characterization of those cells based on developmental lineage and molecular phenotype that has allowed further refinement of their key defining features. By this definition, RTN neurons share a common lineage, emerging from the dB2 domain of rhombomere 5 and expressing transcription factors Egr2, Phox2b, Lbx1, and Atoh1 at various times during early development as they differentiate and migrate to their ultimate destination in the rostral ventrolateral medulla (van der Heijden and Zoghbi, 2020). The intersectional combination of Phox2b and Atoh1 expression selectively identifies just two cell groups in the mouse brainstem: the peri-facial (periVII) neurons comprising the RTN, and a second peri-trigeminal (periV) cell population that controls lapping behavior in mice (Huang et al., 2012; Hirsch et al., 2013; Ruffault et al., 2015; Dempsey et al., 2021). Of the transcription factors associated with RTN development, only Phox2b expression persists at appreciable levels in postnatal RTN neurons; however, Phox2b is also found in other neurons, including the nearby C1 adrenergic neurons and facial motoneurons (Stornetta et al., 2006). Additional work using immunochemical and single cell molecular approaches has produced a more precise and limited phenotypic definition for RTN neurons (Figure 1Aiii) (Shi et al., 2017; Cleary et al., 2021). In addition to Phox2b expression, all RTN neurons express *Slc17a6* (VGlut2); they can be differentiated from other nearby Phox2b-expressing populations, like C1 neurons and motoneurons, by the absence of tyrosine hydroxylase (TH) and choline acetyltransferase (ChAT) expression (Stornetta et al., 2006; Guyenet et al., 2019). All RTN neurons express the excitatory neuropeptide PACAP (pituitary adenylate cyclase activating peptide), and subsets also express variable levels of the inhibitory neuropeptides enkephalin and galanin, but these are not specific for the RTN (Stornetta et al., 2009; Shi et al., 2017; Cleary et al., 2021). Of particular note, RTN neurons can be most definitively identified in this region of the rostroventrolateral medulla by their unique and universal expression of the neuropeptide, Neuromedin B (NMB) (Stornetta et al., 2006; Shi et al., 2017). NMB-positive RTN neurons express a variety of receptors for other neuromodulators, including serotonin (primarily 5-HT2C), substance P (NK1R), orexin (Hcrt1/Hcrt2), and ATP (P2Y12) (Guyenet et al., 2019). Finally, the majority of RTN neurons (>80%) express transcripts for two putative pH sensors, the proton activated G-protein coupled receptor GPR4, and the proton inactivated K_2P_ background K^+^ channel TASK-2 (encoded by *Kcnk5*) (Shi et al., 2017); as discussed below, both GPR4 and TASK-2 have been implicated in mediating pH sensitivity of RTN neurons. Coming full circle, the NMB^+^ cells project to multiple pontine and medullary respiratory regions, including to the DRG and VRG that served as the original defining hodological feature of the RTN (Souza et al., 2023). For these reasons, we now use this constellation of specific features to define these neurons within the parafacial region as the RTN.

### 2.1 Criteria #1 and 2

Several different methods have been used to obtain activation and inhibition of RTN neurons, and these manipulations in turn activate or inhibit respiration in both conscious and anesthetized animals. Inhibition (acute) or ablation (chronic) of the RTN also blunts/abolishes the HCVR, both *in vivo* and *ex vivo*.

The RTN region is crucial for maintaining normal respiration. Acute ablation (via local kainic acid injection or electrolysis) decreases phrenic nerve activity, often to the point of apnea (Nattie et al., 1988; Nattie and Li, 1990), and this nontargeted disruption of the RTN region is also sufficient to abolish the HCVR (Nattie et al., 1988). Selective developmental elimination of the RTN has been achieved using various mouse genetic models (e.g., by Atoh1 deletion in Phox2b cells, inactivation of Phox2b in Atoh1 cells, expression of Phox2b polyalanine expansion or Lbx1 frameshift mutations); this physical deletion of the RTN in turn leads to disrupted baseline breathing in embryos and neonates, and severely blunts CO_2_-evoked breathing stimulation at birth (Dubreuil et al., 2008; Pagliardini et al., 2008; Marina et al., 2010; Patwari et al., 2010; Ramanantsoa et al., 2011; Ruffault et al., 2015; Hernandez-Miranda et al., 2018). Moreover, selective intersectional deletion of VGlut2 from Phox2b-Atoh1 neurons reduces baseline ventilation and eliminates the HCVR in P0 mouse pups. Likewise, essentially complete ablation of the RTN in adults (∼90–95% loss of Nmb^+^ neurons), either by targeted bilateral injection of saporin-conjugated substance P in rats or viral-mediated Cre-dependent expression of caspase in Nmb-Cre mice, reduces baseline breathing (partially compensated by carotid body input) and nearly completely abolishes the HCVR (Figure 1B) (Souza et al., 2018; Souza et al., 2019; Souza et al., 2023).

Transient activation of RTN neurons via photoactivation of channelrhodopsin 2 (ChR2) expressed in RTN neurons under the control of a Phox2b-responsive promotor (PRSx8) increases minute ventilation (V_E_) through effects on both tidal volume and frequency and occludes further activation by CO_2_. These effects are observed in both conscious and anesthetized animals, and ChR2-mediated increases in V_E_ depend on glutamatergic transmission from the RTN (Figure 1C) (Abbott et al., 2009; Abbott et al., 2011; Basting et al., 2015; Holloway et al., 2015; Souza et al., 2020). Conversely, acute inhibition of Phox2b- or Nmb-expressing neurons in the RTN with the inhibitory opsin, ArchT, transiently decreases V_E_ in room air, and silences CO_2_-stimulated RTN neuronal activity and V_E_ (Figures 1D, E) (Basting et al., 2015; Souza et al., 2023). Similarly, inhibition of the RTN with an inhibitory GPCR (*Drosophila* allatostatin receptor) blunts phrenic nerve discharge intensity and frequency at baseline as well as during an acute hypercapnic challenge in an *ex vivo* brainstem-spinal cord preparation (Marina et al., 2010).

### 2.2 Criterion #3

There is good evidence for CO_2_-evoked activation of RTN neurons *in vivo*. For example, neurons in the RTN anatomical region, as well as the molecularly defined Phox2b^+^/NMB^+^ cells, express high levels of the neuronal activity marker Fos after acute hypercapnic challenge (Sato et al., 1992; Teppema et al., 1994; Okada et al., 2002; Kumar et al., 2015; Shi et al., 2017). Direct electrophysiological assessments by extracellular recordings in anesthetized rats *in vivo* and in isolated brainstem-spinal cord preparations have identified neurons within the anatomical boundary of the RTN displaying “respiratory modulated” activity at baseline as well as CO_2_-stimulated activity during hypercapnic challenge (Figure 1F) (Pearce et al., 1989; Connelly et al., 1990; Nattie et al., 1993; Kawai et al., 1996; Mulkey et al., 2004; Guyenet et al., 2005; Stornetta et al., 2006; Marina et al., 2010; Basting et al., 2015). As expected for RTN neurons, the CO_2_-stimulated cells are Phox2b^+^, as demonstrated by *post hoc* immunostaining of the juxtacellularly-labeled recorded neurons (Figure 1F) (Stornetta et al., 2006). The CO_2_-modulated RTN cell firing activity occurs in the absence of feedback from the central pattern generator, i.e., it initiates at a CO_2_ threshold lower than required for phrenic nerve activity and persists after carotid body denervation, glutamate receptor blockade, or pharmacologic silencing of the respiratory central pattern generator (Figure 1F) (Mulkey et al., 2004; Guyenet et al., 2005).

It is important to point out that these *in vivo* electrophysiological recordings were obtained in anesthetized animals, and because anesthetics can exert complex direct and indirect effects on RTN neurons and other respiratory nuclei (Lazarenko et al., 2010a), this leaves open the possibility that the cells might respond differently if recorded in conscious animals. In this respect, indirect measures of RTN neuron function in freely behaving rats are also consistent with CO_2_-modulated neuronal activity. That is, the ventilatory-depressant effects of ArchT-mediated inhibition of RTN neurons are enhanced under conditions of elevated CO_2_ or lower arterial pH, implying that RTN neuronal activity and contribution to respiratory drive is similarly enhanced under those conditions (Figure 1E) (Basting et al., 2015). More recent work applying implanted miniscope imaging of neuronal GCaMP6f dynamics in the region containing the RTN demonstrates the presence of neurons in freely behaving mice that track inspired CO_2_ via graded increases in Ca^2+^ signal, along with other CO_2_-insensitive cells. Whereas these experiments represent an advance in visualizing neuronal activity in a deep medullary structure, like the RTN, those specific chemosensitive cells were not directly targeted and the molecular identity of the recorded neurons was not confirmed. Thus, it remains unclear whether the mixed population that was imaged included the chemosensitive RTN neurons in the region (i.e., Phox2b^+^/Nmb^+^, with GPR4 and/or TASK-2 expression), and it seems certain that the sampling was diluted by recording from the multiple other neuronal subtypes present in the general parafacial region (Bhandare et al., 2022). Future experiments using this technique will undoubtedly use currently available targeting approaches to sample the behavior of specific phenotypically-defined cell populations. Overall, the available evidence provides strong support for the conclusion that RTN neuronal activity tracks with CO_2_/H^+^ *in vivo*, in both anesthetized and conscious animals, even if direct recordings of that activity in freely behaving animals still remain elusive.

### 2.3 Criterion #4

**FIGURE 2:**
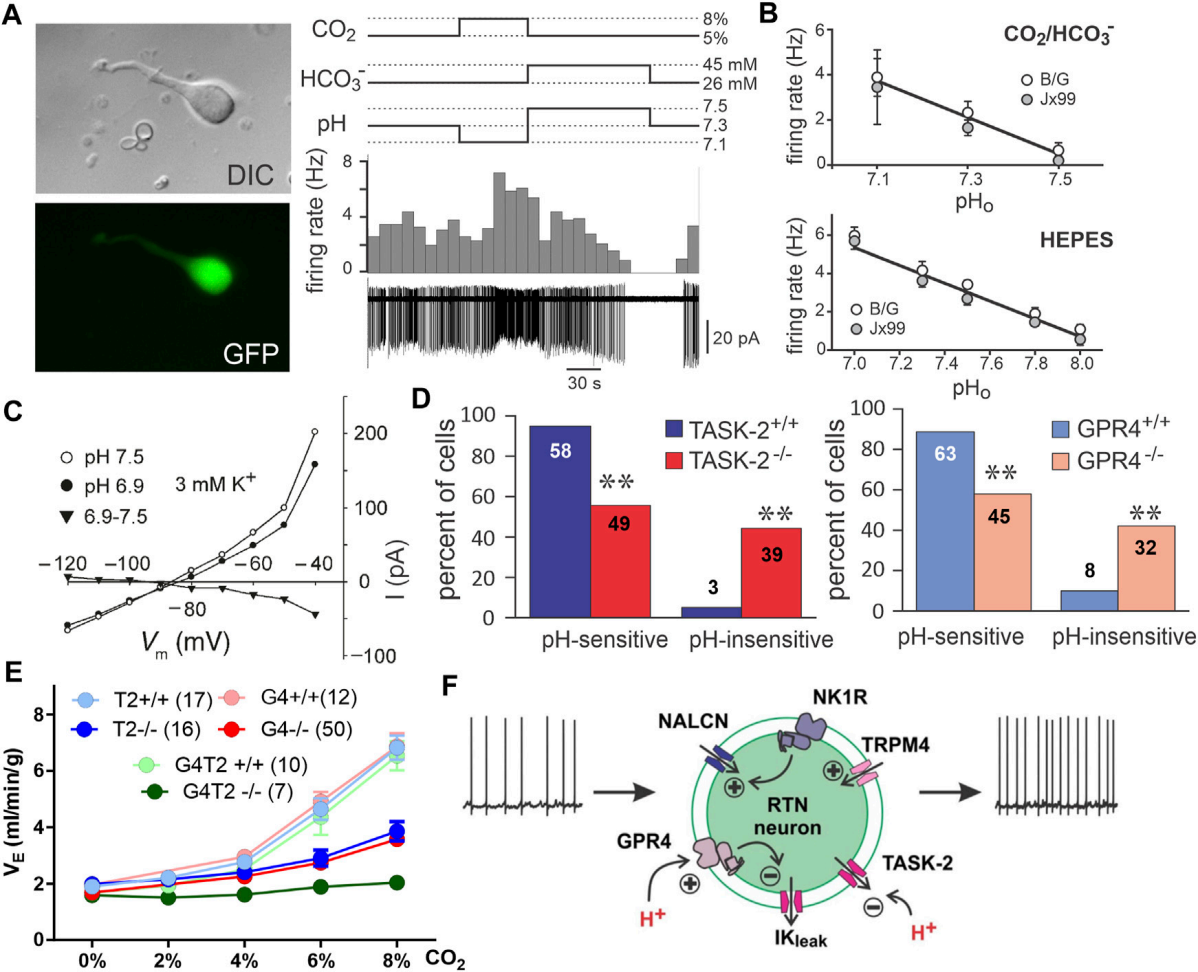
RTN neuron pH sensitivity is intrinsic and mediated by the proton sensors TASK-2 and GPR4. **(A)** Dissociated GFP-positive RTN neurons isolated from Phox2b-GFP mice maintain pH and CO_2_ sensitivity. Panel adapted from (Wang et al., 2013b), Figure 1B, Figure 3A. **(B)** Phox2b-GFP neurons dissociated from two mouse lines maintain pH_o_ sensitivity in HEPES and bicarbonate buffers. Panel adapted from (Wang et al., 2013b), Figures 3C, D. **(C)** RTN neurons demonstrate a pH sensitive K-current in the acute slice treated with TTX. Panel adapted from (Mulkey et al., 2004), Figure 6C. **(D)** Genetic deletion of TASK-2 or GPR4 renders a subpopulation of RTN neurons pH insensitive in slice recordings. Panel adapted from (Wang et al., 2013a), Figure 2E; (Kumar et al., 2015), Figure 2C. **(E)** Whole body knockout of TASK-2 and/or GPR4 blunts (GPR4^−/−^ or TASK-2^−/−^) or nearly eliminates (GPR4^−/−^;TASK-2^−/−^) the HCVR as measured by whole body plethysmography in conscious mice. Panel adapted from (Guyenet et al., 2019), Figure 6G. **(F)** Schematic of RTN neuron illustrating mechanisms mediating tonic firing and K^+^ channel modulation by hypercapnic acidosis. Panel adapted from (Guyenet et al., 2019), Figure 5B.

RTN neurons are intrinsically sensitive to changes in CO_2_/H^+^ across a variety of *in vitro* preparations, including brainstem-spinal cord preparations, acute or cultured brainstem slices and, importantly, acutely dissociated neurons (Figure 2A) (Mulkey et al., 2006; Mulkey et al., 2007a; Lazarenko et al., 2010b; Hawryluk et al., 2012; Wenker et al., 2012; Wang et al., 2013a; Sobrinho et al., 2014; Hawkins et al., 2015; Kumar et al., 2015; Wu et al., 2019). During early development, a group of CO_2_/H^+^ sensitive, Phox2b-expressing neurons in the parafacial region display rhythmic pre-and post-inspiratory firing patterns in brainstem-spinal cord preparations; these have been called the embryonic parafacial oscillator (ePF) or, in the early postnatal period (P0-P2), the parafacial respiratory group (pFRG), and are most likely early precursors to the RTN (Onimaru et al., 2008; Thoby-Brisson et al., 2009; Ruffault et al., 2015). In slightly older neonatal brainstem slice preparations (>P6), RTN neurons are tonically active at physiological pH levels, depolarize and increase action potential firing during bath acidification, and hyperpolarize and decrease firing during bath alkalization. This modulation is observed with changes in fixed acid in HEPES-based buffers and with changes in CO_2_ in HCO_3_^−^-based buffers (Figure 2B); these effects appear to track with changes in extracellular pH since RTN neuron firing is increased by hypercapnic acidosis and reduced by normocapnic alkalosis in CO_2_/HCO_3_^−^-based solutions (Mulkey et al., 2004). The pH sensitivity of RTN neurons is retained in acute slices in the presence of tetrodotoxin (TTX, to block action potential-dependent release) and in low Ca^2+^/high Mg^2+^ synaptic blockade solutions (Mulkey et al., 2004). In addition, pH-dependent modulation of RTN neurons is preserved when slices are exposed to a variety of neurotransmitter receptor blockers, e.g., for glutamate (CNQX, APV), GABA (bicuculline), glycine (strychnine), ATP (suramin, reactive blue 2, PPADS, MRS2179), 5-HT (ketanserin, SB269970), and substance P (spantide, L-703606) (Mulkey et al., 2004; Mulkey et al., 2006; Mulkey et al., 2007a). Finally, individual GFP-positive cells dissociated from the parafacial region of two distinct lines of Phox2b-GFP mice, which were verified as *bona fide* RTN neurons by single cell RT-PCR (i.e., Phox2b^+^, VGlut2^+^, TH-, ChAT-), were also found to retain their CO_2_/H^+^ sensitivity (Lazarenko et al., 2010b; Wang et al., 2013b; Wu et al., 2019). Together, these data make a compelling case that RTN neurons are intrinsically chemosensitive, and they also suggest a molecular basis for direct modulation of neuronal activity by CO_2_/H^+^. However, it should be noted that respiration is exquisitely sensitive to changes in CO_2_, and the effects of CO_2_/H^+^ on RTN firing *in vitro* appear to be quantitatively less robust than those effects *in vivo*, even in anesthetized animals (Guyenet et al., 2005). Thus, whereas direct actions of CO_2_/H^+^ on RTN excitability seem certain, this does not preclude additional indirect effects by modulators that enhance baseline excitability or convey information regarding CO_2_/H^+^ changes that are sensed remotely.

Multiple neurotransmitters, including those that arise from alternative candidate chemoreceptor cells, are known to affect RTN neuronal excitability and may thereby also modulate the firing response to CO_2_/H^+^ (Moreira et al., 2021). This includes serotonin and substance P (from raphe neurons) (Mulkey et al., 2007a), orexin (from the lateral hypothalamus), (Lazarenko et al., 2011), and ATP (from local astrocytes) (Mulkey et al., 2006; Gourine et al., 2010; Wenker et al., 2010). In the case of 5-HT and ATP it has been suggested that these modulators are themselves responsible for conferring an apparent pH sensitivity onto RTN neurons that instead originates from CO_2_/H^+^ sensitive raphe neurons and/or astrocytes (Gourine et al., 2010; Wu et al., 2019). However, the evidence for such an obligatory role of 5-HT and ATP is inconclusive. For example, ketanserin (5-HTR_2_ antagonist) or SB269970 (5-HTR_7_ antagonist) can block RTN activation by exogenous 5-HT *in vitro* (Mulkey et al., 2007a; Wu et al., 2019), but these same blockers are reported in different *in vitro* preparations to either have no effect or abrogate the CO_2_/H^+^ sensitivity of RTN neurons (Mulkey et al., 2007a; Wu et al., 2019). *In vivo*, direct injection of SB269970 into the RTN of conscious mice blocked respiratory stimulation by a co-injected 5-HT_7_ agonist but did not alter CO_2_-stimulated breathing (Shi et al., 2022). Similarly inconsistent results have been obtained with purinergic P2X/Y receptor antagonists (i.e., with suramin, PPADS, MRS2179, reactive blue 2), which have variably been shown to dampen (Gourine et al., 2010; Wenker et al., 2012) or to have no effect on (Mulkey et al., 2004; Mulkey et al., 2006; Mulkey et al., 2007a; Onimaru et al., 2012) CO_2_/H^+^ induced RTN neuronal activity.

Collectively, the available data support the overall conclusion that the CO_2_/H^+^ sensitivity of RTN neurons is a cell intrinsic effect, at least in part. This fulfills criterion #4. It also seems certain that input from other presumptive chemoreceptors (raphe, astrocytes), along with additional modulators from various other cell groups (muscarinic, noradrenergic), can enhance baseline activity of RTN neurons and thereby facilitate their response to CO_2_/H^+^ (Moreira et al., 2021). It is possible that these combined effects—excitatory neuromodulation superimposed on intrinsic CO_2_/H^+^ sensitivity—may account for the difference in CO_2_/H^+^ sensitivity that has been observed between *in vitro* and *in vivo* recordings, and perhaps also for various physiological changes in CO_2_/H^+^ sensitivity (e.g., between sleep and wake states) (Guyenet et al., 2005).

### 2.4 Criterion #5

Under voltage clamp, in the presence of TTX and a cocktail of blockers of fast synaptic transmission, acid-evoked depolarization of the RTN is mediated by inhibition of a pH dependent background K^+^ current (Figure 2C). Activation of RTN neurons by CO_2_/H^+^, as well as full expression of the HCVR, requires the expression and activity of two pH sensitive molecules: TASK-2 and GPR4 (Gestreau et al., 2010; Wang et al., 2013a; Kumar et al., 2015).

TASK-2 is a background K^+^ channel expressed in RTN neurons and in a limited number of additional brainstem cell groups (Gestreau et al., 2010). It shows highest sequence similarity to the TWIK-related alkaline-activated (TALK) subgroup of K_2P_ channels compared to the similarly named, and also pH sensitive, TASK-1 and TASK-3 channels (Lesage and Barhanin, 2011). Inhibitory gating of TASK-2 occurs through the physiological pH range and is mediated via independent intracellular and extracellular pH sensor domains, each with a pH_50_∼8.0 (Niemeyer et al., 2006; Niemeyer et al., 2007; Niemeyer et al., 2010; Lesage and Barhanin, 2011; Li et al., 2020). Inhibition of TASK-2 by acidification leads to membrane depolarization and increased cell excitability. It has not been directly tested whether changes in internal and/or external pH changes account for TASK-2-mediated activation of RTN neurons although, as mentioned above, experimental manipulation of CO_2_ and HCO_3_^−^ levels in bath solutions suggest a primary role for extracellular pH. Whereas nearly all GFP-expressing RTN neurons with wild-type TASK-2 alleles are pH-sensitive in brain slices from Phox2b-GFP mice (∼95%), only 56% of those GFP^+^ RTN neurons are pH-sensitive in TASK-2 deleted mice; the pH-sensitive background K^+^ current is reduced in pH-sensitive cells from these TASK-2 global knockout mice, and eliminated in ∼44% of cells that emerged as pH-insensitive after TASK-2 deletion (Wang et al., 2013a). In TASK-2 global knockout mice, the stimulation of breathing by CO_2_ is strongly reduced (by ∼60% at 8% CO_2_) while baseline respiration is unaffected (Gestreau et al., 2010; Wang et al., 2013a; Kumar et al., 2015). We should note that TASK-2 global knockout mice present with a slight metabolic acidosis (ΔpH: −0.03) (Warth et al., 2004), and it is possible that this could have influenced the HCVR. However, the HCVR is unaffected when a more severe metabolic acidosis is induced chronically in mice by NBCe1 deletion from the kidney (ΔpH: −0.2) (Li et al., 2023), or acutely in human subjects by treatment with carbonic anhydrase inhibitors (ΔpH: −0.1) (Teppema and Dahan, 1999; Teppema et al., 2020).

GPR4 is a proton sensing GPCR expressed in RTN neurons (Kumar et al., 2015; Hosford et al., 2018); it senses extracellular proton concentration via protonation/deprotonation of multiple histidine residues on its outward facing surface (Ludwig et al., 2003; Liu et al., 2010; Tobo et al., 2015). Depending on the expression system it can couple to Ga_s_- and Ga_q_-mediated signaling pathways with a pH_50_ of 7.2–7.6 (Ludwig et al., 2003; Tobo et al., 2007; Liu et al., 2010; Kumar et al., 2015; Tobo et al., 2015; Hosford et al., 2018). In addition to the RTN, GPR4 transcript is also detectable in a limited number of brain nuclei, including the caudal and dorsal raphe nuclei, the lateral septum, and C1, as well as in endothelial cells (Kumar et al., 2015; Shi et al., 2017; Hosford et al., 2018). In the acute slice, treatment with a GPR4 antagonist (Dalton M46; Niemeyer et al., 2010), or whole-body knockout of GPR4, alters the ratio of pH-sensitive to pH-insensitive RTN neurons, with the appearance of a pH-insensitive population that accounts for ∼40% of the recorded cells (Figure 2D) (Kumar et al., 2015). The remaining pH-responsive population of RTN neurons are presumably those that have intact TASK-2-mediated pH sensitivity. CO_2_-dependent activation of RTN neurons *in vivo* (Fos expression) is also reduced in GPR4 global knockout mice while activation of caudal raphe neurons (pallidus, obscurus, magnus, and parapyramidal) is unaffected by GPR4 deletion (Kumar et al., 2015). Administration of the GPR4 antagonist NE 52-QQ57 to mice and rats via an intraperitoneal (i.p.) bolus injection (20 mg/kg) blunts the HCVR by a small, but significant, amount in conscious animals (Hosford et al., 2018). The concentration NE 52-QQ57 reaches at the relevant GPR4-expressing populations after systemic administration is unknown so this inhibition may represent only a small fraction of receptor antagonism *in vivo*. Localized application of NE 52-QQ57 on the ventral surface of the medulla had no effect on the HCVR in anesthetized animals but it is not clear whether the compound reached efficacious levels for GPR4 inhibition at the RTN (Hosford et al., 2018). Importantly, genetic elimination of GPR4 reduced the HCVR (by ∼60% at 8% CO_2_) and selective re-expression of GPR4 in the RTN alone restores CO_2_-induced Fos expression in RTN neurons and rescues the respiratory defects observed in GPR4 global knockout animals (Kumar et al., 2015). This indicates that the expression of GPR4 specifically in RTN neurons may be especially crucial for both RTN neuronal activation and the HCVR. Notably, simultaneous global deletion of both GPR4 and TASK-2 in mice nearly completely abolishes the HCVR (by ∼90% in 8% CO_2_) (Figure 2E) (Kumar et al., 2015), approximating the deficit in HCVR observed with gross ablation of RTN neurons (Souza et al., 2018; Souza et al., 2019; Souza et al., 2023). The effect of RTN-specific deletion of either proton sensor on baseline respiration or the HCVR has not yet been reported.

It is also worth noting that RTN neurons fire action potentials in a steady pacemaker-like pattern both *in vitro* as well as *in vivo*, when other respiratory-related inputs are eliminated (Mulkey et al., 2004; Guyenet et al., 2005; Lazarenko et al., 2010b). The ionic basis for this tonic firing involves a background Na^+^ current, carried by NALCN, and a Ca^2+^-activated cationic current with TRPM4-like properties (Figure 2F) (Shi et al., 2016; Li et al., 2021). These channels contribute to cell excitability, basal activity, and the firing responses to neuromodulators and H^+^. However, neither is directly responsible for intrinsic CO_2_/H^+^ sensing by RTN neurons (Shi et al., 2016; Li et al., 2021). Nevertheless, the HCVR is significantly blunted *in vivo* after either shRNA-mediated knockdown of NALCN, or pharmacological inhibition of TRPM4 in the RTN (Shi et al., 2016; Li et al., 2021). These examples provide a cautionary note: they illustrate how cellular and molecular manipulations that affect general cell function and excitability can modulate the HCVR, even when the targets are not responsible for intrinsic CO_2_/H^+^ sensitivity (i.e., when they are not “sensors”.)

### 2.5 Summary

For the RTN, there is compelling evidence, albeit not yet complete, supporting each of the enumerated criteria. It is clear that positively and negatively modulating RTN neuron activity *in vivo* has the expected effects of facilitating and inhibiting respiration; in addition, RTN neuron activation can occlude effects of CO_2_ on breathing and inhibiting/ablating RTN neurons blunts/eliminates the HCVR. Single unit recordings in anesthetized rats clearly demonstrate CO_2_ modulation of RTN neuronal activity *in vivo*, but only indirect evidence based on Fos expression or implied from effects of ArchT inhibition has been obtained from conscious animals. This particular criterion would be better supported by recordings of activity specifically from RTN neurons in freely behaving animals (e.g., GCaMP for either photometry or single cell imaging of Nmb^+^ cells). It is also clear that RTN neurons are intrinsically sensitive to CO_2_/H^+^, and that this intrinsic sensitivity is imparted by expression of both TASK-2 and GPR4 (Figure 2F). It will be important to understand why intrinsic CO_2_/H^+^-dependent activation, as measured *in vitro*, is less robust than that observed *in vivo*. If this reflects additional neuromodulatory effects *in vivo* from other CO_2_/H^+^-sensitive neuron populations, a better delineation of the relative contributions to overall CO_2_/H^+^-stimulated RTN activity would be helpful. Finally, it remains a formal possibility that TASK-2 and GPR4 are only necessary to maintain excitability of RTN neurons and that pH sensitivity is conferred to those cells through other inputs. This proposition seems unlikely as there are no deficits in baseline respiration of either GPR4 or TASK-2 knockout animals like those that occur with silencing of the RTN via chemo/optogenetic means, indicating that RTN activity remained at or above the threshold necessary to provide baseline respiratory drive in both single and double knockout animals. Importantly, the amino acid determinants of intrinsic pH sensitivity are known for both GPR4 and TASK-2, and so it should be possible to generate genetic models to test whether selective elimination of pH sensitivity, *per se*, is sufficient to recapitulate the observed respiratory effects of the cognate gene knockouts.

## 3 Serotonergic raphe

**FIGURE 3:**
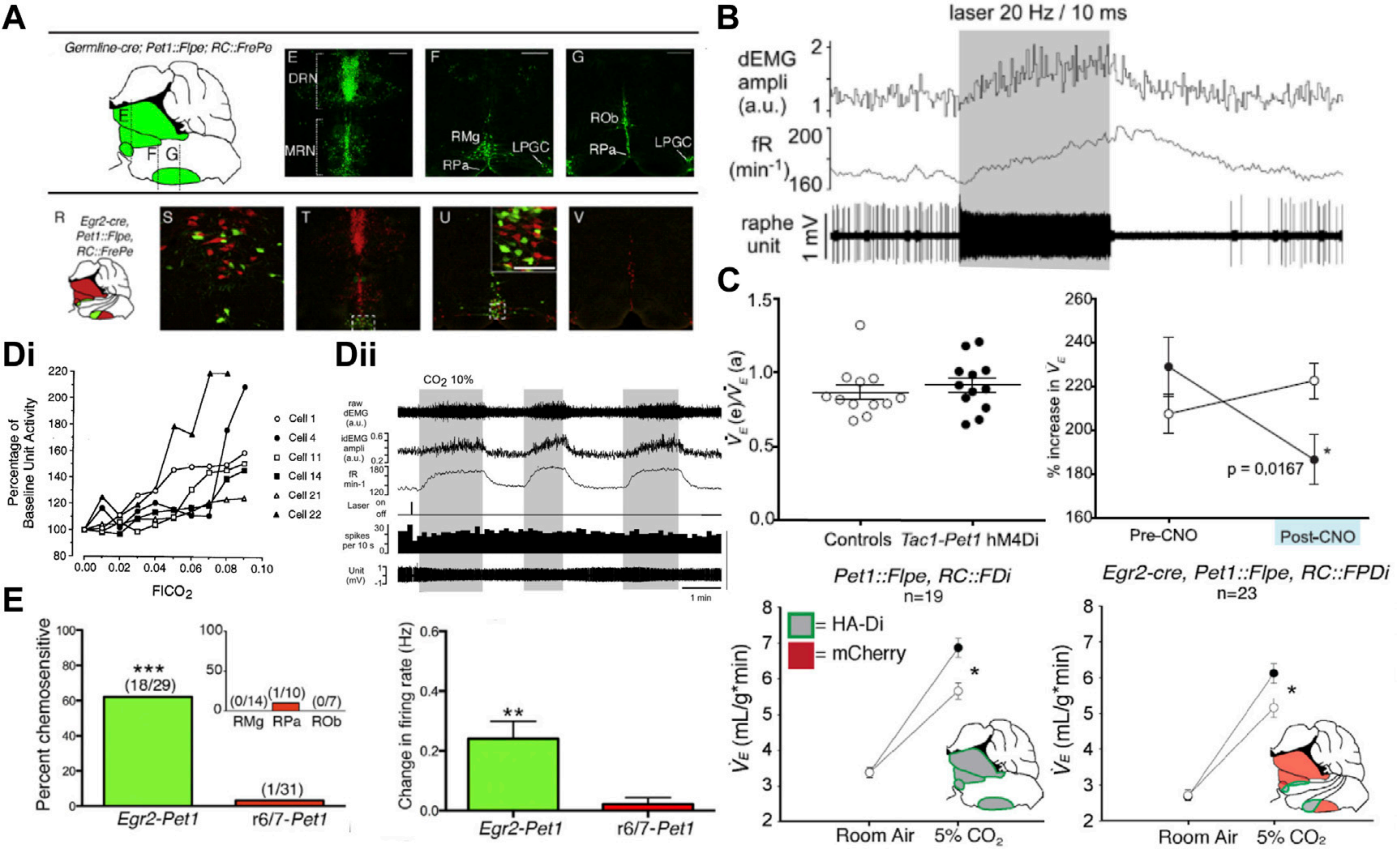
Activation of serotonergic caudal raphe neurons drives respiration and inhibition of the Egr2-Pet1 and Tac1-Pet1 subsets blunts the HCVR. **(A)** Location of Pet1 expressing (top, green) raphe neurons which includes the total population of serotonergic neurons, and the Pet1-Egr2 subset (bottom, green) which comprises the candidate chemoreceptor population. Panel adapted from (Brust et al., 2014), figures 2D–G and 2R-V. **(B)** ChR2-mediated activation of Pet1 expressing midline raphe neurons increases frequency and amplitude of diaphragmatic EMG bursts. Panel adapted from (DePuy et al., 2011), figure 6A1. **(C)** CNO-mediated inhibition of all Pet1+ neurons (lower, left), or the Tac1+ (upper, right) or Egr2+ (lower, right) subpopulations of Pet1+ raphe neurons with an inhibitory DREADD (hM4Di) leads to a blunted HCVR without affecting ventilation in room air. Panel adapted from (Hennessy et al., 2017), Figures 2F, I (top); and (Brust et al., 2014), Figures 3B,E (bottom). **(D)(i)** A subset of raphe obscurus neurons in the conscious cat display increased firing rate in hypercapnia but **(D)(ii)** CO_2_-activated firing is not seen in ChR2 opto-tagged midline Pet1 expressing raphe neurons in anesthetized rat. Panel adapted from (Veasey et al., 1995), Figure 7 (left, copyright 1995 Society for Neuroscience) and (DePuy et al., 2011), Figure 7A (right). **(E)** CO_2_ sensitive firing in Egr2-Pet1 expressing raphe pallidus neurons in the acute slice under conditions of synaptic block (picrotoxin, AP-5, and CNQX); intermingled r6/7-derived Pet1+ caudal raphe cells were not CO_2_-sensitive. Panel adapted from (Brust et al., 2014), Figures 4E, K.

The brainstem raphe nuclei include the dorsal raphe (DR), median raphe (MnR), raphe magnus (RMg), raphe pallidus (RPa), raphe obscurus (ROb), and the parapyramidal (PPy) cell groups; they contain all the serotonergic neurons in the CNS, along with other non-serotonergic neurons. Among the serotonergic raphe neurons there is a wide diversity of neuronal subtypes as defined by both developmental origin and molecular phenotype (Figure 3A). Elegant recent work has meticulously catalogued these serotonergic neuron subtypes while also providing a range of intersectional genetic tools that have begun to find use in probing differential functions of raphe neurons (Brust et al., 2014; Hennessy et al., 2017; Okaty et al., 2019; Okaty et al., 2020; Senft et al., 2021).

Among raphe nuclei, the medullary serotonergic neurons have been most clearly implicated in respiratory function; they target a number of respiratory cell groups including the RTN, preBötC, NTS, LC, lPBN, hypoglossal motor nucleus, and motor neurons in the ventral horn of the spinal cord (e.g., phrenic motoneurons) (Connelly et al., 1989; Smith et al., 1989; Thor and Helke, 1989; Holtman et al., 1990a; Holtman et al., 1990b; Manaker and Tischler, 1993; Hoökfelt et al., 2000; Ribas-Salgueiro et al., 2005; Mulkey et al., 2007a; Ptak et al., 2009; DePuy et al., 2011; Brust et al., 2014). Similar to the other candidate nuclei discussed in this review, microdialysis of low pH/high CO_2_ equilibrated aCSF into different raphe nuclei increases respiration and/or arousal (Nattie and Li, 2001; Hodges et al., 2004; Smith et al., 2018). In addition, when applied intracerebroventricularly *in vivo* or in bath perfusion solutions *in vitro*, 5-HT can activate multiple distinct respiratory-related nuclei, including the RTN (Holtman et al., 1984; Bayliss et al., 1995; Bayliss et al., 1997a; Bayliss et al., 1997b; Peña and Ramirez, 2002; Schwarzacher et al., 2002; Mulkey et al., 2007a; Hodges et al., 2008; Ptak et al., 2009; Hawryluk et al., 2012; Hawkins et al., 2015; Wu et al., 2019). Pharmacological approaches have also been used to decode the roles of different 5-HT receptors in control of breathing. Bath treatment of a brainstem spinal cord preparation with an agonist of inhibitory 5-HT_1A_ autoreceptors leads to complete loss of CO_2_-stimulated phrenic nerve discharge (Corcoran et al., 2013). Inhibition of 5-HT_2_ receptors in acute slice or brainstem-spinal cord preparations leads to blunted respiratory outflow (i.e., decreased XII or phrenic nerve discharge amplitude/frequency) (Peña and Ramirez, 2002; Brandes et al., 2006; Hodges et al., 2009; Ptak et al., 2009; Corcoran et al., 2013). Reciprocally, 5-HT_2_ receptor activation increases motor outflow (Cayetanot et al., 2002; Schwarzacher et al., 2002; Hodges et al., 2009). These effects are proposed to occur via 5-HT activation of receptors in the RTN, preBötC and various respiratory motor nuclei. This evidence is generally consistent with a role for the serotonergic system in modulating respiration, including CO_2_-stimulated breathing, and provides the rationale for additional experiments with greater cell-type selectivity that address potential mechanisms for CO_2_/H^+^ detection by serotonergic neurons.

### 3.1 Criteria 1 and 2

The specific contribution of serotonergic raphe neurons to respiration and the HCVR has been examined by gross ablation approaches and by using chemogenetic or optogenetic tools. Mice with developmental depletion of serotonergic neurons exhibit a blunted HCVR and impaired CO_2_-induced arousal along with an inability to regulate body temperature during a thermal challenge (Hodges et al., 2008; Hodges et al., 2009; Smith et al., 2018). Acute chemotoxic ablation of SERT^+^ or NK1R^+^ neurons in the midline raphe causes a decrease in basal respiration as well as a blunted HCVR, mainly through decreases in tidal volume (V_T_) (Nattie et al., 2004). More recently, cell-specific chemogenetic or optogenetic approaches have assessed the effect of acute raphe activation or inhibition on respiration at rest and during a hypoxic or hypercapnic challenge. For example, after viral-mediated Cre-dependent expression of ChR2 in Epet-Cre mice, optogenetic activation of ROb neurons increased the frequency and amplitude of diaphragmatic EMG in anesthetized mice (Figure 3B); in the presence of CO_2_, the respiratory stimulation by ChR2 activation of ROb neurons was proportional to the CO_2_-elevated baseline respiratory output (DePuy et al., 2011). Conversely, inhibition of the SERT-Pet1, Egr2-Pet1 and Tac1-Pet1 neuron subpopulations of neurons via intersectional expression of the hM4Di inhibitory DREADD and subsequent CNO administration blunted the HCVR without any effect on baseline respiration (Figure 3C) (Ray et al., 2011; Brust et al., 2014; Hennessy et al., 2017). These data provide strong support for the conclusion that serotonergic raphe neuronal activity can modulate respiratory output and the HCVR, satisfying the first two criteria.

### 3.2 Criterion 3

There is evidence for activation of serotonergic raphe neurons by CO_2_ *in vivo*, but it remains somewhat ambiguous. Support for CO_2_-induced raphe neuron activation has been obtained by using indirect proxy measures, such as changes in 5-HT levels or Fos expression after *in vivo* exposure to CO_2_ (Sato et al., 1992; Larnicol et al., 1994; Johnson et al., 2005; Kanamaru et al., 2007; Iceman et al., 2013; Kumar et al., 2015). However, direct measures of CO_2_ effects on raphe firing activity *in vivo* have yielded inconsistent results—and opposing conclusions. For example, early extracellular recordings from the 1980s in unanesthetized cats supported the idea that the activity of raphe neurons can be modulated by CO_2_ *in vivo* (Veasey et al., 1995; Veasey et al., 1997). Veasey, Jacobs and others found a subset of recorded neurons from both dorsal (8/36) and caudal (6/27) raphe neurons displayed firing activity that tracked with inspired CO_2_; moreover, firing activity in CO_2_ correlated impressively with increased V_E_ (Figure 3Di) (Veasey et al., 1995; Veasey et al., 1997). Notably, the CO_2_ sensitivity of caudal raphe neurons was state-dependent (absent in slow wave sleep), even though that is a period when respiration is strongly dependent on chemoreceptor input. In addition, the activity of all CO_2_-sensitive caudal raphe neurons increased during motor activity (treadmill locomotion), consistent with a general role in motor function. Although the neurochemical phenotype of these recorded neurons was not definitively established, the physiological, pharmacological, and functional characteristics, together with their anatomical location, indicate that they were likely serotonergic. Overall, these early data suggested that only a subset of serotonergic raphe neurons is CO_2_ sensitive, and they are thus consistent with the recent recognition of multiple genetically, developmentally, and functionally diverse subgroups of serotonergic raphe neurons in mice (Okaty et al., 2019; Okaty et al., 2020).

**FIGURE 4:**
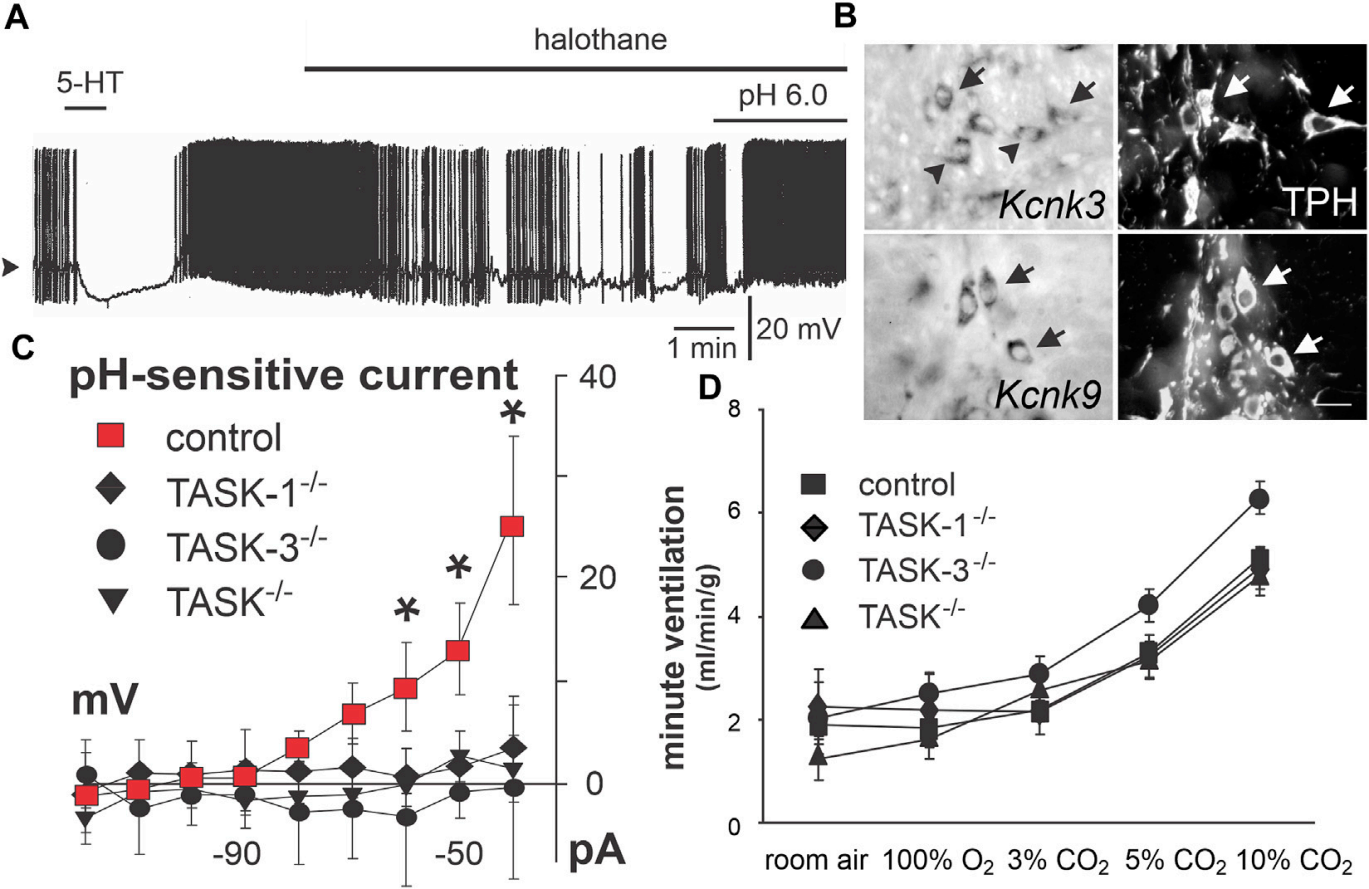
TASK-1 and TASK-3 expression underlies a TASK-like pH- and halothane-sensitive K^+^ current in raphe neurons but is not required for CO_2_-stimulated breathing. **(A)** Acidosis stimulated activity of caudal raphe neurons persists under halothane inhibition. Panel adapted from (Washburn et al., 2002), Figure 7C, copyright 2002 Society for Neuroscience. **(B)** TASK-1 (Kcnk3) and TASK-3 (Kcnk9) expression in caudal raphe serotonergic (Tph+) neurons. Panel adapted from (Washburn et al., 2002), Figure 4, copyright 2002 Society for Neuroscience. **(C)** Dorsal raphe neurons demonstrate a pH sensitive whole cell current that is lost in TASK-1 and TASK-3 single or double knockout animals. Panel adapted from (Mulkey et al., 2007b), Figure 3D, copyright 2007 Society for Neuroscience. **(D)** Whole body plethysmography shows no effect of single or double knockout of TASK-1 and/or TASK-3 on the HCVR in freely behaving mice. Figure adapted from (Mulkey et al., 2007b), Figure 6B, copyright 2007 Society for Neuroscience.

In subsequent work, direct electrophysiological recordings of CO_2_ stimulated activity in rodent raphe neurons *in vivo* have been sought, but not yet obtained. For example, in halothane- or isoflurane-anesthetized rats (n = 37 cells, N = 4 rats) and mice (n = 20, N = 4), neurons recorded in medullary raphe nuclei (ROb, RPa, Ppy) were generally insensitive to increases in inspired CO_2_ (Figure 3Dii) (Mulkey et al., 2004; DePuy et al., 2011). These neurons showed functional characteristics expected of serotonergic neurons, and this was further verified either by juxtacellular labeling and *post hoc* tryptophan hydroxylase (TPH) immunostaining in rats (Mulkey et al., 2004) or by opto-tagging following ChR2 expression in ePet-cre mice (DePuy et al., 2011). It is notable that despite their CO_2_ insensitivity, optogenetic activation of spontaneously active, ChR2-expressing serotonergic raphe neurons was able to stimulate respiratory output in a manner proportional to the effects of CO_2_. As mentioned, raphe neurons are functionally diverse, and it is possible that the neurons sampled in these experiments did not include the subset of CO_2_-sensitive cells that were identified, perhaps fortuitously, in the cat raphe. It is also possible that *in vivo* CO_2_ sensitivity of raphe neurons is affected by anesthesia. This possibility is supported by experiments using an *in situ* unanesthetized decerebrate rat brainstem preparation in which CO_2_-sensitive serotonergic raphe neurons were identified, and for which subsequent exposure to isoflurane caused membrane hyperpolarization and inhibited spontaneous and CO_2_-evoked firing (Massey et al., 2015). Although it was suggested that raphe neuron inhibition by anesthesia could be due to anesthetic activation of pH-sensitive TASK-1/TASK-3 channels, which are enriched in serotonergic raphe neurons, it is important to point out that proton-mediated inhibition of those TASK channels is maintained, and actually enhanced, in the presence of anesthetics (Figure 4A) (Hirshman et al., 1977; Patel et al., 1999; Sirois et al., 2000; Talley et al., 2001; Massey et al., 2015). Thus, any CO_2_/H^+^-sensitivity attributable to those TASK channels would be retained even in the presence of halothane or isoflurane.

At this point, support for CO_2_ sensitivity of serotonergic raphe neuron activity *in vivo* remains equivocal; further *in vivo* recordings, directed specifically toward the subgroup of putative chemosensitive raphe neurons (i.e., Egr2-Pet1 cells) and perhaps incorporating GCaMP-enabled fiber photometry or cell imaging, would be particularly helpful in resolving the question of the chemosensitivity of serotonergic raphe neurons in freely behaving animals *in vivo*. The latter was recently attempted; miniscope recordings of GCaMP6s-expressing serotonergic neurons in RMg and RPa of conscious mice uncovered multiple types of CO_2_-dependent responses (e.g., transient activation, inhibition), with a graded response to CO_2_ observed in some cells (8/26) (Bhandare et al., 2022).

### 3.3 Criteria 4 and 5

Despite the ambiguous results from *in vivo* studies described above, it is abundantly clear from extensive experiments that medullary raphe neurons are directly activated by CO_2_/H^+^ *in vitro*; this has been repeatedly demonstrated in the acute slice, in slice culture and, importantly, under conditions of fast synaptic blockade and/or in dissociated neurons where indirect activation is precluded (Richerson, 1995; Wang et al., 1998; Richerson et al., 2001; Wang et al., 2002; Severson et al., 2003; Richerson et al., 2005; Brust et al., 2014; Massey et al., 2015). The CO_2_/H^+^-sensitivity is preferentially in the serotonergic (TPH^+^/Pet1^+^) population within raphe cells recorded *in vitro*, and recent work indicates that this property appears to be specific to the Egr2-Pet1-expressing subset of serotonergic neurons (Figure 3E) (Brust et al., 2014).

The molecular substrate(s) for modulation of serotonergic raphe neuron activity by CO_2_/H^+^ have not been definitively identified, and thus criterion #5 has not been conclusively tested for these putative respiratory chemoreceptors. Nevertheless, some candidates merit discussion. The anesthetic-activated and pH-sensitive background K^+^ channels TASK-1 and TASK-3 are expressed at high levels in serotonergic raphe neurons throughout postnatal development (Figure 4B), and a corresponding anesthetic- and pH-sensitive background current was observed in recordings from serotonergic neurons of the dorsal and caudal raphe *in vitro* (Talley et al., 2001; Washburn et al., 2002); importantly, this current is eliminated in TASK-1/TASK-3 double knockout mice (Figure 4C), as is the effect of pH on firing in caudal raphe neurons (Mulkey et al., 2007b). Notably, global knockout of these TASK channels has no effect on the HCVR (Figure 4D) (Mulkey et al., 2007b; Trapp et al., 2008). So, if these channels are responsible for the pH sensitivity of these cells, then this result is not consistent with a role for serotonergic raphe neurons in the HCVR. Some caveats are worth noting. The widespread expression of TASK-1/TASK-3 in all serotonergic raphe neuron cell groups (∼80%) does not align with the proposed selective CO_2_ sensitivity limited to only the Egr2-Pet1-expressing subset of neurons in the caudal raphe. In addition, the recordings of TASK channel-dependent pH sensitivity in raphe neurons were obtained in slices from neonatal knockout mice and it is possible that some alternative molecular proton detector mediates CO_2_ sensitivity in adult animals (despite the continued TASK expression) and/or compensates for loss of TASK channels in knockout mice. One potential alternative is GPR4, one of the proton detectors in RTN neurons that is also expressed in serotonergic raphe neurons (Kumar et al., 2015; Hosford et al., 2018). However, there is no functional evidence for a GPR4 contribution to pH modulation of raphe neuron excitability *in vitro*, GPR4 expression is not necessary for CO_2_-induced raphe activation *in vivo* (as assessed by Fos expression), and the inhibition of the HCVR in GPR4-deleted mice is fully rescued by GPR4 re-expression limited only to the RTN (Kumar et al., 2015). It is also worth mentioning that the molecular determinants for pH sensitivity of both TASK-1/TASK-3 channels and GPR4 are localized to extracellular domains, and pH modulation of raphe neuron firing is thought to involve changes in intracellular pH (Wang et al., 2002), leaving open the possibility for another, still unidentified proton detector in raphe neurons. In this regard, it was suggested that the pH dependent current and depolarization in medullary raphe neurons may be due to a novel pH-sensitive and Ca^2+^-dependent nonselective cation channel (Richerson, 1995; Wang et al., 2002; Mulkey et al., 2007a; Trapp et al., 2008). However, no molecular candidate has yet been revealed that fits those still preliminary and unpublished electrophysiological observations.

### 3.4 Summary

There is substantial evidence supporting a role for medullary serotonergic raphe neurons in driving respiratory output and supporting the HCVR, and this now appears to be a function selectively of both the CO_2_/H^+^-sensitive Egr2-Pet1 and presumably CO_2_/H^+^-insensitive Tac1-Pet1 cell subtypes of serotonergic neurons in ROb and RPa (Brust et al., 2014; Hennessy et al., 2017). There is no consensus yet as to whether these cells are activated by CO_2_ *in vivo* but there is strong support for direct activation of serotonergic neurons by CO_2_/H^+^ in reduced preparations; this is true of dorsal and caudal raphe neurons, but within the caudal raphe, it appears that this may be a property selective for the Egr2-Pet1-expressing subset of serotonergic neurons in RPa (Brust et al., 2014). Future experiments should take advantage of the intersectional genetic approaches now available to record specifically from this subset of neurons *in vivo*. Among proposed molecular substrates for the intrinsic CO_2_/H^+^ sensitivity of raphe neurons, the clearest evidence supports a role for TASK-1/TASK-3 channels, at least *in vitro*. Notably, even as genetic deletion of those TASK channels in mice eliminated pH sensitivity in neonatal raphe neurons, loss of TASK channels had no effect on the HCVR in adult mice, a finding inconsistent with a role for serotonergic neurons as respiratory chemoreceptors. However, those data cannot rule out a compensatory mechanism in the knockout mice, or an alternative mechanism in adult mice. As yet, no alternative molecular CO_2_/H^+^ sensor has been identified. The ability to use intersectional approaches to selectively mark the putative subgroup of chemosensitive medullary serotonergic raphe raises the possibility for a differential transcriptomic analysis that may uncover further candidate sensor molecules to examine in the context of addressing the crucial criterion #5 for these cells (Okaty et al., 2015).

## 4 Medullary astrocytes

Astrocytes were historically considered to provide a simple supporting role for neuronal function but work over the last several decades has made it abundantly clear that they are much more intimately involved in shaping neural activity. Astrocytes respond to various physicochemical factors and neuromodulators via intracellular calcium signaling and, in turn, they can regulate the activity of neighboring astrocytes and neurons by secreting various gliotransmitters (e.g., ATP). In the context of respiratory control by CO_2_, the possibility that astrocytes could serve as respiratory chemoreceptors initially derived from the observations that ATP release is stimulated by CO_2_/H^+^ in brainstem regions conventionally associated with chemoreception (Gourine et al., 2005); ATP can drive respiration via actions on P2 purinergic receptors (Gourine et al., 2003); and inhibition of P2 receptors can blunt the effect of CO_2_ on respiratory output (Mulkey et al., 2006; Wenker et al., 2012; Sobrinho et al., 2014; Barna et al., 2016). In addition to metabolic support and direct gliotransmitter actions on nearby brain cells, astrocytes are also well poised to modulate local blood flow during metabolic/respiratory challenge via close apposition of their end feet to CNS vessels (Gourine et al., 2010; Wenker et al., 2010; Hawkins et al., 2017). In brainstem chemoreceptor regions of the ventral medullary surface (VMS), where CO_2_-dependent astrocytic ATP release has been measured, hypercapnia causes a P2-dependent vasoconstriction that decreases the rate of washout of CO_2_ and allows for further activation of chemosensitive neurons and astrocytes (Kasymov et al., 2013; Mishra et al., 2016; Hawkins et al., 2017; Cleary et al., 2020; Marina et al., 2020; Wenzel et al., 2020; Hosford et al., 2022). Together, these observations suggest the presence of distinct populations of astrocytes in different brainstem regions and multiple mechanisms by which the CO_2_/H^+^ sensitivity of astrocytes could translate into enhanced respiratory output. For the purposes of this review, we will discuss these different astrocytic populations and/or cellular mechanisms, noting the anatomical regions in which the functional characterization has been examined.

### 4.1 Criteria 1 and 2

Studies of the effect of exogenous activation of astrocytes have focused on the astrocytes in the region near the RTN and/or the preBötC. Parapyramidal astrocytes have not been specifically targeted for exogenous activation or inhibition experiments. For a number of experiments, spatial delineation was not fine enough to enable distinction between different astrocyte populations.

**FIGURE 5:**
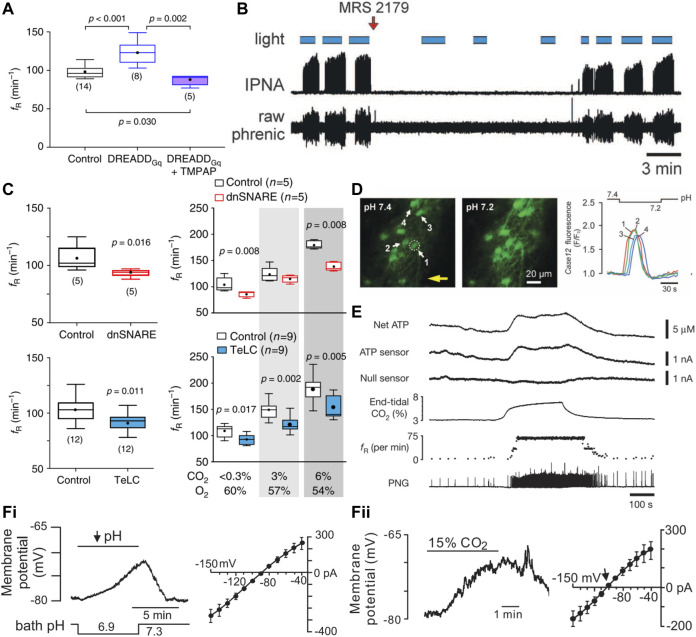
During hypercapnic acidosis, ventral medullary astrocytes are activated, ATP levels increase, and purinergic signaling is necessary for astrocyte activation to drive respiration. **(A)** Activation of preBötC astrocytes with a Gq-coupled DREADD leads to an increased frequency of respiration (fR) in the conscious mouse in an ATP dependent manner (i.e., increase is not present with co-expression of endonucleotidase TMPAP). Panel adapted from (Sheikhbahaei et al., 2018), Figure 2K. **(B)** Activation of astrocytes in the RTN region using ChR2 leads to increased phrenic nerve discharge in a purinergic dependent manner (MRS 2179, P2Y receptor antagonist). Panel adapted from (Gourine et al., 2010), Figure 4C. **(C)** Inhibition of preBötC astrocytic vesicular release using a dominant negative SNARE (dnSNARE) or tetanus toxin (TeLC) expression leads to decreased fR at baseline and during exposure to elevated CO_2_. Panel adapted from (Sheikhbahaei et al., 2018), Figures 1G, H, 4B. **(D)** Astrocytes on the ventral surface of a medullary slice in the RTN region respond to low pH by increasing intracellular Ca^2+^. Panel adapted from (Gourine et al., 2010), Figure 1B. **(E)** ATP levels increase on the ventral surface of the brainstem in isolated heart brainstem preparation perfused with high CO_2_ solution. Panel adapted from (Gourine et al., 2005), Figure 1A. **(F)** Whole cell recordings of astrocytes in RTN region of rat brain slices show membrane depolarization and development of a weakly-rectifying CO_2_/H^+^ sensitive current during bath acidification **(i)** or exposure to elevated CO_2_ **(ii)**. Panel adapted from (Wenker et al., 2010), Figures 1A, G, Figures 3A, E.

Optogenetic ChR2-mediated stimulation of ventral medullary astrocytes in an *ex vivo* preparation (likely containing the RTN area) drives astrocytic calcium transients (Gourine et al., 2010); in brain slice culture, ChR2 stimulation of the same astrocytes leads to depolarization and increased firing of nearby RTN neurons (identified via expression of Phox2b) (Gourine et al., 2010). Additionally, ChR2 activation of VMS astrocytes in anesthetized rat increases phrenic discharge and can drive phrenic activity during hypocapnic apnea (Gourine et al., 2010). The induction of RTN firing as well as phrenic discharge resulting from ChR2 excitation of VMS astrocytes is blocked by MRS2179, a P2Y receptor antagonist (Figure 5B) (Gourine et al., 2010). It is important to note the caveat that ChR2 stimulation of astrocytes can lead to elevations in extracellular K^+^, which may contribute to depolarization and firing of RTN neurons, although direct K^+^-dependent neuronal excitation would not be sensitive to P2Y receptor blockers. In addition, depolarization of RTN-adjacent astrocytes with fluorocitrate, a putatively astrocyte selective metabolic disrupter, can increase the firing frequency of individual RTN neurons in slices, and increase phrenic discharge and respiratory frequency during CO_2_ exposure in anesthetized animals (Erlichman et al., 1998; Holleran et al., 2001; Erlichman and Leiter, 2010; Wenker et al., 2010; Sobrinho et al., 2017). There have been no experiments reported on the effects of acute, transient inhibition of RTN area astrocytes on basal respiration or the HCVR.

Chemogenetic activation of preBötC astrocytes with the excitatory Gq-coupled DREADD increases the respiratory rate in conscious animals in an ATP-dependent manner (Figure 5A) (Sheikhbahaei et al., 2018). A variety of molecular approaches were developed and applied to disrupt astroglial signaling in the preBötC; these include viral-mediated expression in astrocytes of proteins that block vesicular release mechanisms (i.e., dominant-negative SNARE, tetanus toxin subunit) or ATP signaling (i.e., ectonucleotidase) (Sheikhbahaei et al., 2018). Notably, these manipulations of preBötC astrocytes interfere with respiratory responses to multiple stimuli (hypoxia, hypercapnia, exercise), consistent with a general contribution to maintaining respiratory network function. These experiments have not been repeated for the specialized subset of CO_2_/H^+^-activated VMS astrocytes that are proposed to mediate respiratory chemosensitivity.

### 4.2 Criterion 3

Astrocyte activation is typically assayed using fluorescent probes that assess increases in intracellular calcium (e.g., GCaMP, Ca^2+^-sensitive dyes). Ca^2+^ imaging of “ventral surface astrocytes” *in vivo* in the anesthetized rat and *ex vivo* in the acute horizontal slice during an acute pH challenge (HEPES 7.45 → 7.25) reveals a marked increase in astrocytic Ca^2+^ throughout the ventral surface of the brainstem, regions including the RTN and PPy groups of astrocytes (Figure 5D) (Gourine et al., 2010). ATP is proposed to be a primary mediator for astrocytic control of breathing and ATP levels indeed increase at two locations on the VMS in anesthetized rats exposed to CO_2_, albeit by different magnitudes (Figure 5E). The level of ATP released from the area around the facial motor nucleus, containing the RTN, in response to a hypercapnic challenge is significantly lower than that from the parapyramidal area just caudal to the hypoglossal nerve root, possibly reflecting different magnitudes or mechanisms of CO_2_/H^+^ sensitivity (Gourine et al., 2005; Huckstepp et al., 2010a). Neither Ca^2+^ dynamics nor ATP release from preBötC astrocytes during different respiratory challenges have been reported. Note that ATP release is a less satisfying surrogate of astrocyte activation since ATP can also be released from neurons and microglia, and the ATP sensors employed do not provide the spatial/temporal resolution available with Ca^2+^ imaging techniques. A demonstration of astrocytic response to CO_2_/H^+^ in the RTN, cPPy, and/or preBötC regions containing the proposed astrocyte chemoreceptors of the intact, unanesthetized animal, perhaps by measuring Ca^2+^ activity (with GCaMP) or even ATP release with cellular sensors (iATPSnFR, GRAB_ATP_), will be required to better satisfy criterion 3.

### 4.3 Criteria 4 and 5

**FIGURE 6:**
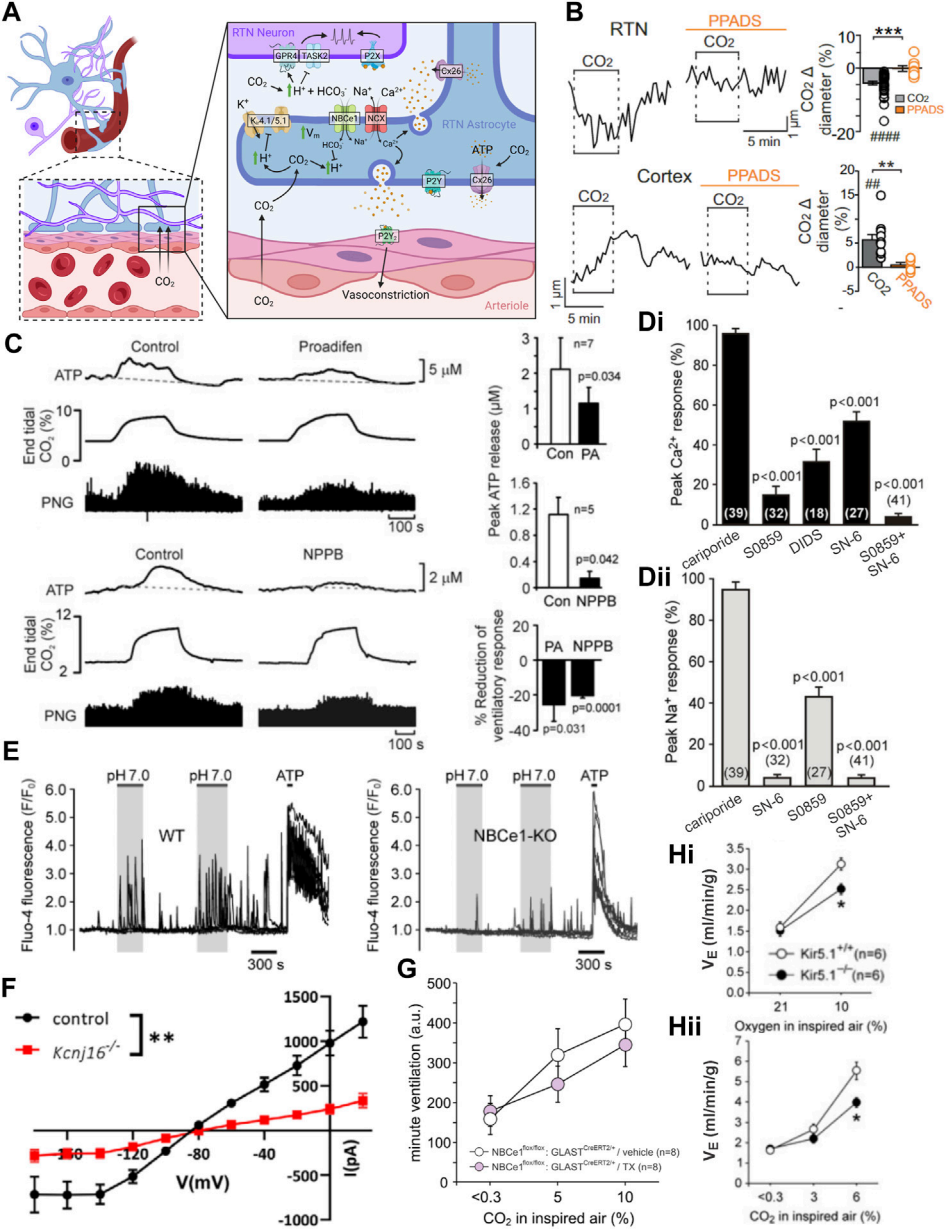
Multiple mechanisms are proposed for astrocyte CO_2_ sensing, CO_2_-dependent ATP release, and effects on CO_2_-stimulated breathing. **(A)** Schematic of proposed astrocytic mechanisms of CO_2_/H+ sensitivity and physiological signaling. Figure prepared in Biorender. **(B)** Medullary vessels in brain slices vasoconstrict in response to CO_2_ and this vasoconstriction is inhibited by a P2 receptor blocker (PPADS). The reverse is observed in cortical vessels. Panel adapted from (Hawkins et al., 2017), Figure 1A, 3A. **(C)** Treatment of the ventral medullary surface with connexin blockers in anesthetized rats attenuates but does not abolish CO_2_ induced phrenic discharge. Panel adapted from (Huckstepp et al., 2010a), Figure 13. **(D)** Blockers of NBCe1 and/or NCX activity blunt pH induced Ca^2+^ **(i)** and/or Na^+^ **(ii)** transients in astrocytes in organotypic slice culture. Panel adapted from (Turovsky et al., 2016), Figure 2F. **(E)** Whole body knockout of NBCe1 reduces the frequency of pH induced Ca^2+^ transients in cultured astrocytes. Panel adapted from (Turovsky et al., 2016), Figures 5A, B. **(F)** RTN astrocyte pH sensitive current is decreased in K_ir_5.1 (*Kcnj16*) knockout animals. Panel adapted from (Patterson et al., 2021), Figure 7F. **(G)** Loss of astrocytic NBCe1 via tamoxifen induced GLAST-Cre driven deletion does not affect the HCVR. Panel adapted from (Hosford et al., 2022), sup. Figure 6B. **(H)** Both the HVR **(i)** and HCVR **(ii)** are significantly blunted in K_ir_5.1 whole body knockout mice. Panel adapted from (Trapp et al., 2011), Figures 1A, B.

Multiple mechanisms for activation of distinct populations of VMS astrocytes by CO_2_/H^+^ have been proposed in the context of respiratory chemosensitivity. Astrocytes near the RTN depolarize in response to both low external pH and high CO_2_ exposure (Figure 5Fi, ii) (Wenker et al., 2010), with these VMS astrocytes broadly displaying an increase in intracellular calcium and sodium (Gourine et al., 2010; Turovsky et al., 2016). The proposed molecular bases for these astrocytic CO_2_/H^+^ responses include: a) direct activation of connexin 26 (Cx26) by molecular CO_2_ in astrocytes of the parapyramidal region; b) activation of a Na/HCO_3_^−^ exchanger (NBCe1) by CO_2_-mediated intracellular acidification of preBötC and RTN astrocytes; and c) direct inhibition of K_ir_4.1/5.1 by intracellular H^+^, leading to depolarization of astrocytes adjacent to the RTN (Figure 6A).

In these cases, the physiological coupling from astrocytes to nearby respiratory-related neurons is thought to occur via paracrine purinergic signaling following CO_2_/H^+^-stimulated ATP release and/or by altering the local CO_2_/H^+^ concentration around respiratory chemosensory neurons. These two general effects are not mutually exclusive and can theoretically occur in parallel. For example, released ATP may directly activate respiratory-related neurons while also provoking a local vasoconstriction to reduce washout of metabolic byproducts (e.g., CO_2_); likewise, HCO_3_^−^ uptake due to activation of NBCe1 by intracellular acidification or depolarization can remove buffering equivalents from the extracellular space and further accentuate acidification of the extracellular space. Note that VMS astrocytes are unique compared to other CNS populations in that they induce vasoconstriction, opposed to dilation, of nearby vessels during hypercapnia, likely via a P2Y2-dependent mechanism (Figure 6B) (Kasymov et al., 2013; Mishra et al., 2016; Hawkins et al., 2017; Cleary et al., 2020; Marina et al., 2020; Wenzel et al., 2020; Hosford et al., 2022). Here, we describe these three proposed molecular CO_2_/H^+^ sensors, the associated physiological coupling mechanisms, and outline the brainstem regions where they have been examined for a role in respiratory chemosensitivity *in vivo*.

#### 4.3.1 Activation of Cx26 in parapyramidal astrocytes

Connexins can form hemichannels capable of mediating release of the gliotransmitter ATP from astrocytes; together with its breakdown product ADP, these signaling molecules are proposed to act on nearby VMS respiratory neurons and vasculature to control the chemoreflex. Pharmacological inhibition of connexins decreases the level of ATP released on the VMS during a hypercapnic event in the anesthetized and ventilated rat (Figure 6C). The same inhibition also blunts the effect of 10% CO_2_ on respiration but does not affect baseline respiration in anesthetized/ventilated rats (Huckstepp et al., 2010a). Based on its expression pattern, Cx26 was proposed to be the most likely connexin conduit for ATP release from VMS/preBötC astrocytes (Solomon et al., 2001a; Solomon et al., 2001b). Subsequently, heterologous Cx26 expression was found to confer CO_2_ sensitivity to non-chemosensitive cells, and Cx26 was shown to be gated by molecular CO_2_ (not H^+^) via a lysine carbamylation event (Huckstepp et al., 2010a; Huckstepp et al., 2010b; Meigh et al., 2013; Dospinescu et al., 2019). Development of a dominant negative Cx26 (dnCx26) allowed for lentiviral-based manipulation of Cx26 carbamylation in subsets of VMS astrocytes at various levels along the respiratory column to assess effects on respiration (van de Wiel et al., 2020). It should be recognized that these experiments represent a test of the final and most stringent criterion we have outlined—i.e., examining effects on the HCVR of disrupting a molecular CO_2_-sensing mechanism in the relevant cells. In fact, expression of dnCx26 in the caudal parapyramidal area (cPPy) reduced the V_T_ component of the HCVR at 6% CO_2_ and was noted only at the level of the cPPy, not rostrally near the RTN or more caudal to the cPPy (van de Wiel et al., 2020). However, respiratory effects were limited only to changes in V_T_, were observed at a single intermediate CO_2_ concentration (not at 3% or 9% CO_2_) and did not persist for all timepoints tested. The effects of dnCx26 expression on CO_2_-evoked ATP release in the cPPy were not reported (Meigh et al., 2013).

#### 4.3.2 Activation of NBCe1 in the RTN and/or preBötC areas

Astrocytes express high levels of NBCe1, an electrogenic Na^+^-HCO_3_^-^ co-transporter that buffers acid-base changes associated with high levels of neuronal activity. In this proposed model of medullary astrocyte activation (Turovsky et al., 2016), elevated CO_2_ leads to an increase in intracellular [H^+^] which drives import of HCO_3_^−^ through NBCe1 to buffer changes in intracellular pH. The concomitant increase in intracellular Na^+^ leads to the reversal of a Na^+^-Ca^2+^ exchanger (NCX), providing the Ca^2+^ uptake required for vesicular release of ATP. Aside from initiating ATP release, the uptake of HCO_3_^−^ can remove buffering equivalents from the extracellular space, potentially exacerbating local acidification. Consistent with this mechanism, the CO_2_-dependent Ca^2+^/Na^+^ signal in astrocytes is completely blocked *in vitro* by the NBCe1 inhibitor S0859 and partially blocked by inhibition of NCX (Figure 6Di, ii) (Turovsky et al., 2016). In addition, deletion of NBCe1 from medullary astrocytes decreases the frequency of acid induced Ca^2+^ transients (Figure 5E) (Turovsky et al., 2016). Although this cellular mechanism is well documented *in vitro*, recent work from NBCe1 knockout mice has failed to support a role for astrocytic NBCe1 in the HCVR. That is, no significant difference in the HCVR was observed in multiple conditional knockout models in which recombination of floxed NBCe1 alleles was achieved in astrocytes by using GFAP-Cre, Aldh1l1-CreERT2 and GLAST-CreERT2 mouse lines (Figure 6F) (Hosford et al., 2022; Li et al., 2023). In the GFAP-Cre line, consistent with GFAP expression patterns, NBCe1 deletion was particularly prominent in astrocytes along the ventral medullary surface, including near the RTN (Hosford et al., 2022); the more widespread NBCe1 deletion obtained with the two tamoxifen-inducible CreERT2 lines was obtained in adults, avoiding potential issues with developmental compensation (Hosford et al., 2022; Li et al., 2023). Although these data suggest that astrocytic NBCe1 expression is not required for the HCVR, it remains possible that elimination of NBCe1 was incomplete in these models and/or spared some select population of astrocytes that are involved in chemosensation.

#### 4.3.3 Inhibition of K_ir_4.1/5.1 channels in RTN area astrocytes

Astrocytes display inwardly rectifying K^+^ currents that have been attributed to K_ir_4.1/5.1 heteromeric channels; these channels are directly inhibited by a decrease in intracellular pH such as occurs during a hypercapnic challenge in medullary astrocytes (Figure 6G) (Tanemoto et al., 2000; Xu et al., 2000; Pessia et al., 2001; Patterson et al., 2021; Zhang and Guo, 2023). Inhibition of the K^+^ channel causes astrocytic depolarization, promoting NBCe1-mediated Na^+^ and HCO_3_^−^ uptake due to the electrogenic nature of the transporter (Wenker et al., 2010; Mulkey and Wenker, 2011). As described above, the associated Na^+^ influx and reversal of NCX can provide the increased intracellular Ca^2+^ import for vesicular release of ATP. In support of this model, preliminary observations suggested a reduced V_T_ response to CO_2_ in astrocyte-specific K_ir_4.1 knockout mice (Hawkins et al., 2014), and whole body knockout of the gene coding for K_ir_5.1 leads to profound metabolic acidosis and blunting of the HCVR and HVR (Figure 6Hi, ii) (Trapp et al., 2011; Puissant et al., 2019). The blunted chemoreflex in Kir5.1 mice could reflect chemoreflex desensitization due to sustained metabolic acidosis rather than any specific effect of K_ir_5.1 deletion on chemosensing by astrocytes (Trapp et al., 2011). Moreover, as noted below, there is also evidence for expression of K_ir_5.1 in LC neurons, which have been separately implicated in respiratory chemosensitivity.

### 4.4 Summary

There is abundant evidence from *ex vivo* and *in vivo* preparations showing that increases in CO_2_/H^+^ can drive calcium signaling in astrocytes and provoke release of ATP in multiple regions associated with respiratory chemosensitivity, at least in part from astrocytes. A demonstration of astrocytic activation by CO_2_ has not yet been realized in unanesthetized animals. It has also been demonstrated that ChR2 activation of VMS astrocytes can activate RTN chemosensitive neurons and stimulate breathing, likely via P2Y (and possibly P2X) receptors. Notably, there is disagreement over the necessity for purinergic stimulation in CO_2_/H^+^ activation of RTN neurons, but it seems likely that engagement of P2 receptors plays some role. The approaches used to inhibit astrocyte signaling in the preBötC support a contribution to the HCVR but those same manipulations also affect respiratory stimulation by a number of other stimuli. Moreover, they have not yet been applied in the ventral medullary regions where astrocytes were proposed to regulate CO_2_/H^+^ sensitivity via nearby RTN neurons (Gourine et al., 2010). Although this is consistent with broadly distributed respiratory chemosensory function, it is also possible that this reflects a relatively non-specific support of neuronal activity. Indeed, interpretations of experiments disrupting astrocyte activity in terms of specific chemosensory functions are complicated by the baseline functions of astrocytes in regulating K^+^ buffering, neurotransmitter recycling/release, synaptic function, local blood flow, etc., and by the fact that modulation of the HCVR by astrocytes must ultimately be channeled through neurons within respiratory circuits. Finally, there is evidence for at least three different molecular mechanisms for CO_2_ and/or H^+^ sensing by distinct populations of astrocytes throughout the medulla. It is unknown whether these groups of medullary astrocytes differ in their reliance on any particular sensory mechanism. To date, *in vivo* tests of each mechanism in the context of the HCVR have yielded results that are either inconclusive (e.g., K_ir_4.1/5.1 inhibition) or do not support a necessary role (Cx26 carbamylation, NBCe1 activation). For K_ir_ channels, studies that eliminate their function specifically in astrocytes would be helpful. It is also possible that these mechanisms are redundant during hypercapnia *in vivo*, and that simultaneous inhibition of more than one mechanism is necessary to uncover some more prominent role. Finally, alternative molecular mechanisms for proton sensing by astrocytes may yet be uncovered.

## 5 Locus coeruleus

The locus coeruleus (LC) is a brainstem structure located in the rostral pons, lateral and ventral to the fourth ventricle; it comprises ∼3000 noradrenergic neurons in mouse or rat, providing the primary noradrenergic innervation throughout the central nervous system (Loizou, 1969; Schwarz and Luo, 2015; Liu et al., 2021; McKinney et al., 2023). Its activity is tightly correlated to arousal levels and stress. Neurons within the LC are electrically coupled via gap junctions, particularly in their dendritic processes, and they exhibit a steady pacemaker-like baseline firing pattern with a pronounced subthreshold oscillation (Ishimatsu and Williams, 1996; Oyamada et al., 1999; Ballantyne et al., 2004). Like the serotonergic raphe, the LC is historically considered a part of the “reticular activating system” and, as such, it influences the activity of many targets in an arousal-state-dependent manner. There is considerable evidence that LC neurons can influence respiration and display intrinsic CO_2_/H^+^ sensitivity, but the most powerful of the new technical advancements in neuroscience have not yet been applied to addressing the significant gaps in fulfilling the criteria that would be necessary for acceptance as *bona fide* central respiratory chemoreceptors.

### 5.1 Criteria 1 and 2

**FIGURE 7:**
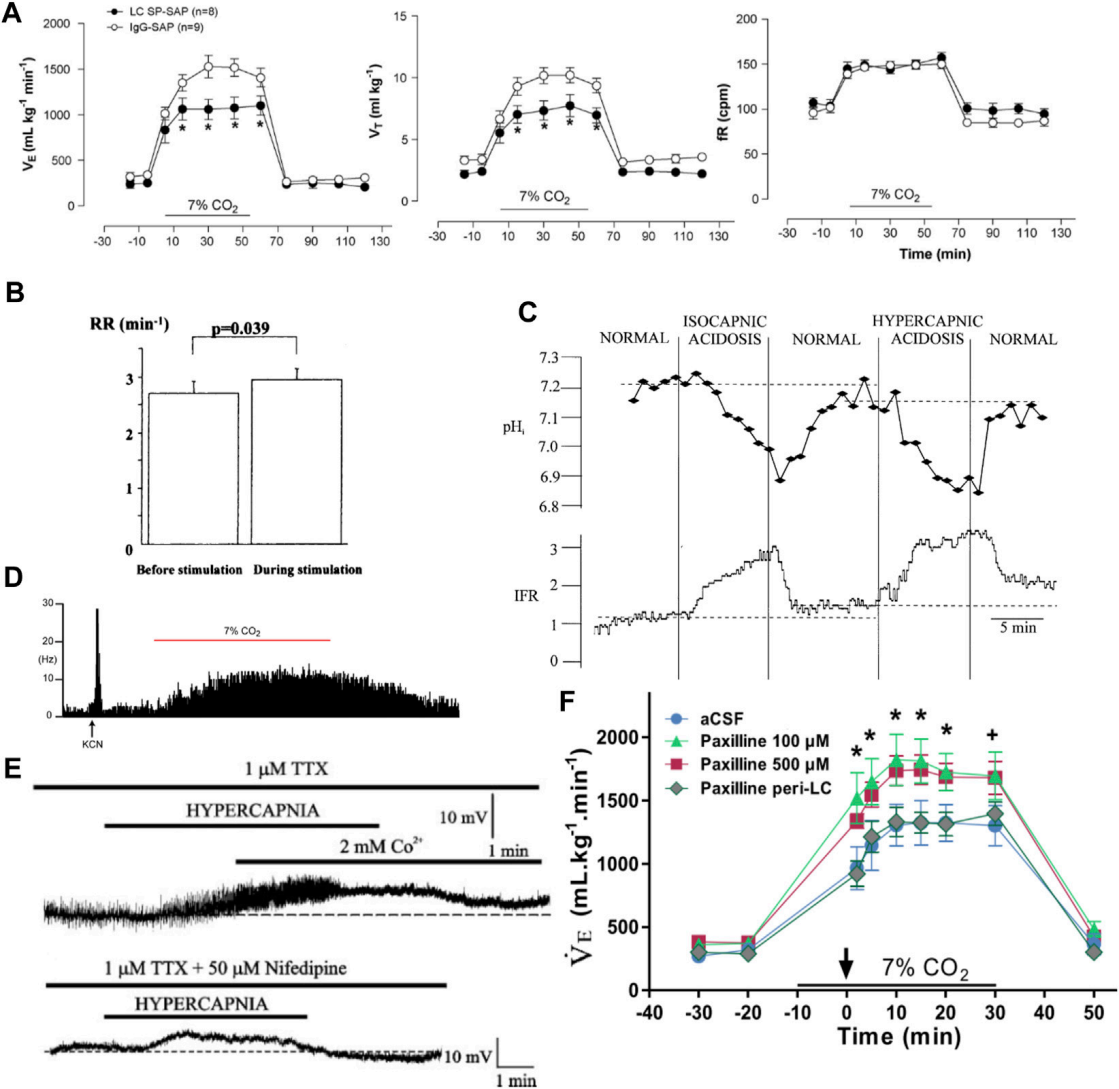
Locus Coeruleus neurons are activated by CO_2_ and they contribute to a normal HCVR. **(A)** Targeted ablation of LC neurons using SP-saporin leads to a blunted HCVR through altered CO_2_ effects on tidal volume and not frequency. Panel adapted from (de Carvalho et al., 2010), Figure 4. **(B)** Electrical stimulation of the LC in the isolated brainstem spinal cord preparation leads to a small increase in hypoglossal nerve burst frequency (respiratory rate, RR). Panel adapted from (Hakuno et al., 2004), Figure 7A. **(C)** In rat brainstem slices, LC neuronal activity (integrated firing rate, IFR) tracks internal pH and does not require changes in CO_2_. Panel adapted from (Filosa et al., 2002), Figure 7. **(D)** In extracellular recordings from anesthetized rat, LC neuron firing increases during hypoxia (KCN, potassium cyanate) and hypercapnia (7% CO2). Panel adapted from (Magalhães et al., 2018), Figure 1C. **(E)** In rat brainstem slices, LC neurons exhibit a subthreshold membrane potential oscillation that increases in power and frequency during hypercapnic acidosis (see thickening and darkening of trace); the oscillation is blocked by extracellular Co^2+^ and nifedipine (L-type Ca^2+^ channel blocker). Panel adapted from (Filosa and Putnam, 2003), Figure 3. **(F)** Injection of BK inhibitor paxilline directly into the LC to remove the oscillatory brake does not affect baseline respiration but augments the HCVR. Panel adapted from (Imber et al., 2018), Figure 9B.

The effects of LC activation and inhibition on respiration and the HCVR have been tested in several ways, mostly indirect. Targeted ablation of the LC via injection of SP-saporin or anti-DBH-saporin decreases the magnitude of the HCVR without effects on basal respiration in room air (Figure 7A) (Li and Nattie, 2006; de Carvalho et al., 2010). Microinjection of the carbonic anhydrase inhibitor acetazolamide to produce a local acidification in the LC caused an increase in phrenic nerve output in anesthetized cats and rats (Coates et al., 1993). Likewise, microinjection of agonists of purinergic signaling or antagonists of serotonergic or glutamatergic signaling into the LC augment the HCVR via effects on tidal volume (De Moreno et al., 2010; Biancardi et al., 2014). Similar targeted injection of antagonists of purinergic, orexinergic, or gap junctional activity attenuate the whole body HCVR predominantly through effects on tidal volume (De Moreno et al., 2010; Taxini et al., 2013; Biancardi et al., 2014; Patrone et al., 2014; Vicente et al., 2016). Acute electrical excitation of the LC in an *ex vivo*, brainstem spinal cord preparation can increase C4 burst frequency, albeit by a small amount (Figure 7B) (Hakuno et al., 2004). In a more direct test, acute inhibition of the LC via activation of an exogenously expressed inhibitory allatostatin receptor has no effect on baseline respiration but this was able to blunt the ventilatory response to 7% CO_2_ (Magalhães et al., 2018). Analogous experiments using chemo or optogenetics to determine the effects of acute LC activation on respiration in the conscious, behaving animal have not yet been reported.

### 5.2 Criterion 3

Animals exposed to an acute hypercapnic challenge display increased levels of Fos in the LC (Haxhiu et al., 1996; Teppema et al., 1997; Berquin et al., 2000). LC firing activity tracks both hypercapnia and hypoxia in the anesthetized rat *in vivo*, without relying on peripheral chemoreceptor input (Figure 7D) (Elam et al., 1981; Magalhães et al., 2018).

### 5.3 Criteria 4 and 5

There is good evidence for direct chemosensitivity of LC neurons *in vitro*. For example, LC neurons increase action potential firing in response to a hypercapnic challenge *in vitro*; this is likely a direct effect of CO_2_/H^+^ since it is resistant to synaptic block with kynurenic acid, picrotoxin, or low Ca^2+^/high Mg^2+^ solution and, importantly, is retained in LC neurons studied after acute dissociation (Ito et al., 2004; Ritucci et al., 2005; Johnson et al., 2008; Nichols et al., 2008; Erlichman et al., 2009). The cellular and ionic bases for CO_2_-dependent changes in cell excitability and action potential firing have been explored extensively. In contrast to the RTN, LC activity tracks internal and not external pH (Figure 7C) (Pineda and Aghajanian, 1997; Filosa et al., 2002; Hartzler et al., 2008). The pH sensitive K_ir_4.1/5.1 channels are expressed in LC neurons at relatively high levels, at least when compared to other candidate chemoreceptor cell groups, and genetic deletion of K_ir_5.1 attenuates the LC firing response to NH_4_Cl-induced internal acidification *in vitro* (Wu et al., 2004; D’Adamo et al., 2011). The expression of K_ir_4.1/K_ir_5.1 in both LC neurons and astrocytes, where they are also implicated as potential pH sensors, confounds interpretation of physiological experiments performed in whole animal K_ir_5.1 knockouts and further supports a move to cell type specific manipulations moving forward. The L-type calcium channels that contribute to subthreshold oscillations and action potential firing are not directly affected by internal pH and LC neurons still depolarize in response to CO_2_ when oscillations are blocked with nifedipine (Figure 7E) (Filosa and Putnam, 2003; Imber and Putnam, 2012; Li and Putnam, 2013; Imber et al., 2014; Imber et al., 2018; Li et al., 2021). However, the subthreshold oscillations are modulated by cAMP/PKA signaling and can be indirectly facilitated by CO_2_-dependent intracellular acidification, uptake of bicarbonate and activation of a bicarbonate-sensitive soluble adenylate cyclase (sAC) (Imber et al., 2014). In addition, calcium-activated K^+^ channel (BK) activity acts as a brake on CO_2_-activated firing in LC neurons (Imber et al., 2018), but this contribution of BK channels to the cellular response appears to be distinct from any role as direct sensors for CO_2_/H^+^.

Even as these mechanisms for CO_2_/H^+^ regulation of LC neuron activity have been examined *in vitro*, their role in initiating or supporting the whole animal HCVR is relatively unknown. As mentioned earlier in the discussion of astrocytes, global genetic deletion of K_ir_5.1 can blunt the HCVR, but it is not possible to attribute this effect to an action on the LC. It has been demonstrated that microinjection of paxilline, a BK inhibitor, into the LC of the adult rat can augment the HCVR via effects on tidal volume, presumably by removal of the oscillatory brake (Figure 7F) (Imber et al., 2018). The effects of targeted inhibition of the other identified components of pH sensitivity in the LC have not yet been reported.

### 5.4 Summary

There is good evidence that the firing activity of LC neurons can be modulated by CO_2_/H^+^ *in vitro*. Aside from the chemogenetic experiment with allatostatin, most of the evidence for *in vivo* modulation of respiration and the HCVR lacks cell specificity and would benefit from application of more targeted optogenetic/chemogenetic approaches. The effects of CO_2_ on LC activity have been observed *in vivo* under anesthesia, but have not yet been examined in freely behaving animals. Finally, despite identification of various ion channel contributors to CO_2_/H^+^-dependent firing in LC neurons, a principal molecular candidate for the intracellular pH sensor has not been forthcoming, and it has therefore not been possible to examine effects of disrupting direct CO_2_/H^+^ sensing in LC neurons on the HCVR or respiration generally.

## 6 Lateral hypothalamus

**FIGURE 8:**
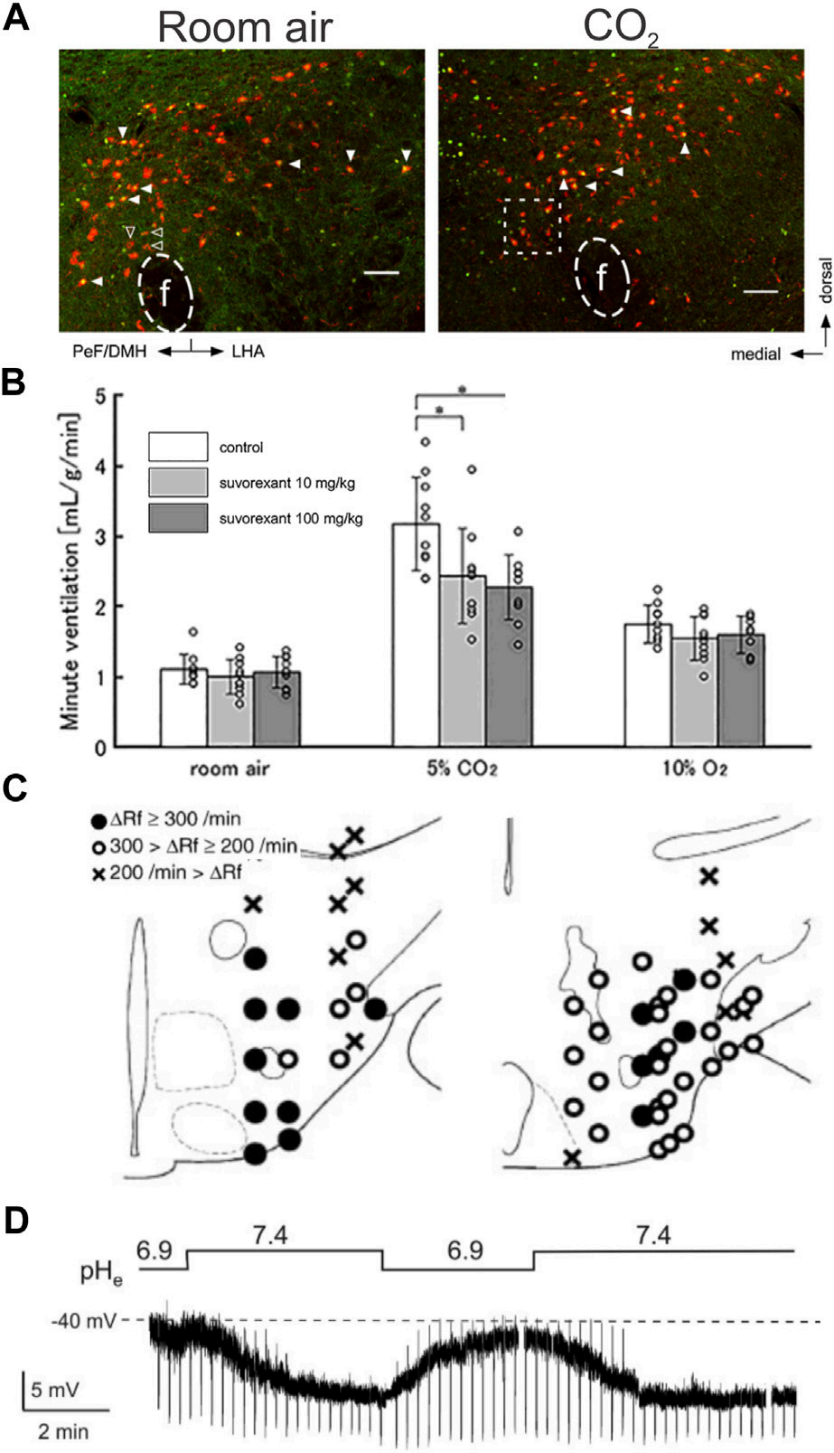
CO_2_/H^+^ can activate orexin-expressing lateral hypothalamus neurons and blocking orexin receptors reduces the HCVR. **(A)** Orexin expressing (red) lateral hypothalamus cells in rat express increased levels of Fos (green) after an acute CO_2_ challenge. Panel adapted from (Sunanaga et al., 2009), Figures 1A, B. **(B)** Treatment with the orexin receptor antagonist suvorexant slightly blunts the HCVR in conscious mice. Panel adapted from (Fukushi et al., 2022), Figure 5A. **(C)** Locations in lateral hypothalamus where electrical stimulation increased respiratory frequency in the anesthetized mouse. Panel adapted from (Kayaba et al., 2003), Figure 1. **(D)** Orexin neuron membrane potential is sensitive to bath pH changes in acute slices from rat under TTX treatment. Panel adapted from (Williams et al., 2007), Figure 4B, copyright 2007 Society for Neuroscience.

The lateral hypothalamus (LH) is a highly heterogeneous region which contains a large proportion of the orexin producing neurons within the CNS. The orexin system has been a focus of recent research on arousal state, cardiorespiratory control, and environmental stress response. The orexinergic neurons in the LH have a broad range of targets throughout the brain, including to the RTN, LC, raphe, and preBötC (Peyron et al., 1998; Trivedi et al., 1998; Date et al., 1999; Nambu et al., 1999; Marcus et al., 2001; Young et al., 2005; Rosin et al., 2006; Puskás et al., 2010; Lazarenko et al., 2011; Tupone et al., 2011; Nattie and Li, 2012). Application of exogenous orexin to a number of these nuclei increases their firing activity and excitability and leads to an altered cardiorespiratory state (Shirasaka et al., 1999; Machado et al., 2002; Zhang et al., 2005; Corcoran et al., 2010; Lazarenko et al., 2011; Young et al., 2005; Shahid et al., 2011; Luong and Carrive, 2012; Shahid et al., 2012; Sugita et al., 2014; Loiseau et al., 2019). Of relevance to central chemoreception, animals exposed to a hypercapnic challenge show increased Fos expression in the orexin cells of the LH (Figure 8A), perfusion of the LH with low pH solution increases respiration rate in an orexin dependent manner, and the ventilatory response to CO_2_ is attenuated in orexin knockout mice (Kayaba et al., 2003; Nakamura et al., 2007; Sunanaga et al., 2009; Song et al., 2012; Li et al., 2013). Treatment with pharmacological antagonists of orexin receptors, which theoretically mediate the downstream effects of orexinergic neuron activation, leads to a blunted HCVR in the whole animal and decreased effects of CO_2_ on phrenic nerve activity in an isolated rat brainstem spinal cord preparation (Figure 8B) (Dias et al., 2009; Corcoran et al., 2010; Li and Nattie, 2010; Vicente et al., 2016; Fukushi et al., 2022). Together, these data support a role for the orexinergic system in respiratory control and provide a rationale to assess the evidence supporting orexinergic LH neurons for their potential roles as respiratory chemoreceptors.

### 6.1 Criteria 1 and 2

The orexin neurons of the LH are interspersed with a number of other cellular subtypes in the LH. Disinhibition of LH neurons via microapplication of the GABA_A_ receptor antagonist bicuculline or direct activation via electrical stimulation leads to an increased heart rate, blood pressure, and frequency of respiration (Figure 8C) but these cell activation methods do not specifically target orexin neurons in this heterogenous area (Kayaba et al., 2003; Iigaya et al., 2012). There are no reports describing the effect of inhibition of LH orexin neurons on baseline respiration or the HCVR.

### 6.2 Criterion 3

As mentioned above, animals exposed to an acute hypercapnic challenge demonstrate increased expression of the activity marker Fos in the orexin neurons of the LH. Other measures of activity during respiratory challenge *in vivo* have not yet been reported for the orexinergic LH neurons.

### 6.3 Criteria 4 and 5

The activity of orexin neurons in acute slices of the LH is modulated by CO_2_/H^+^, and pH mediated depolarization is maintained in the presence of TTX suggesting a cell-intrinsic sensitivity (Figure 8D) (Williams et al., 2007; Song et al., 2012). The molecular mechanism(s) controlling this intrinsic CO_2_/H^+^ modulation are largely uncharacterized. LH orexin cells express high levels of pH sensitive potassium channels TASK-1/TASK-3; those channels regulate cell excitability in orexin neurons but are not necessary for their CO_2_ sensitivity (Gonzalez et al., 2009; Guyon et al., 2009). Respiration can be activated by microinjection into the LH of an extremely low pH solution (pH 6.5); this effect relies on acid-sensitive ion channels (ASICs) but it is unknown if this requirement is due to altered chemosensitivity or just due to overall decreased excitability as seen in the TASK-1/TASK-3 knockout system (Song et al., 2012). The properties of ASIC channels seem poorly suited to homeostatic regulation of ventilation, particularly their pH sensitivity in extreme acidic ranges typically associated with pathophysiology (pH 6-7); moreover, global knockout of ASIC1, ASIC2 or ASIC3 had no effect on HCVR in conscious, unrestrained mice (Guyenet et al., 2016; Detweiler et al., 2018).

### 6.4 Summary

There is good evidence that the orexinergic system can provide a general excitatory drive to respiratory circuits, likely via orexin signaling and in an arousal state-dependent manner. However, the evidence addressing the criteria required for a *bona fide* respiratory chemosensory function is less well developed. Experiments examining effects on respiration and the HCVR using cell-specific methods for activating and inhibiting orexin neurons would be helpful. In addition, although orexin neurons appear to be sensitive to CO_2_ *in vivo* (Fos), like most of the other cell groups reviewed here, there have been no direct measures of this CO_2_-mediated neuronal activation in freely behaving animals. It also seems certain that orexin neurons in the LH can be activated by CO_2_/H^+^ *in vitro*, likely directly, but the cellular and ionic mechanisms so far suggested for intrinsic chemosensitivity of those neurons have not held up to experimental scrutiny, at least in the context of CO_2_-regulated breathing. Thus, better satisfying a number of these criteria, especially identifying and manipulating a relevant molecular CO_2_/H^+^ sensor, will be crucial to support a role for these cells as chemosensors.

## 7 Conclusion

There has been a long-term quest to identify the brainstem sensory cells that detect changes in CO_2_/H^+^ and drive the respiratory circuits that adjust ventilation to correct deviations from normal physiological set points for PaCO_2_ and tissue acid-base balance. As cellular candidates have emerged, there have been additional efforts to use various technical advances to define those cell types with greater phenotypic clarity, seek molecular substrates for their CO_2_/H^+^ sensitivity, and validate their physiological role in respiratory chemosensitivity. To formalize evaluation of these ongoing efforts, we have enumerated a set of increasingly stringent criteria that we believe are necessary and, for the final criterion sufficient, to declare a candidate as a *bona fide* respiratory chemoreceptor (Guyenet and Bayliss, 2022).

Here, we examined the extant experimental support for the most prominent current chemoreceptor candidates and can confidently conclude that none have yet surpassed the full evidentiary bar demanded by these criteria. However, for a number of these cell types we could identify strong, albeit partial, support for many of the criteria.

In the case of the developmentally and biochemically defined RTN neurons, experimental modulation of their activity has the expected effects on respiratory output, and they are directly responsive to CO_2_/H^+^ *in vitro* via two identified proton detectors (TASK-2, GPR4) that are both required for full elaboration of the HCVR. The CO_2_/H^+^ modulation of RTN *in vivo* remains to be directly observed in unanesthetized animals, and the genetic elimination of TASK-2 and GPR4 was global and did not disrupt the pH sensing mechanism, *per se*. Nonetheless, both RTN ablation and combined TASK-2/GPR4 knockout eliminate the HCVR nearly completely in conscious animals, consistent with a particularly prominent role for both RTN neurons and their molecular pH sensors. The effect of RTN ablation also suggests that these neurons may be a point of convergence for inputs from other presumptive chemoreceptors. Indeed, RTN neurons are modulated by several transmitters and peptides from those other cell groups, and such a convergent action may support the more pronounced CO_2_/H^+^ sensitivity of RTN neurons *in vivo*, by comparison to *in vitro*.

The other chemoreceptor candidates that have accrued the most experimental support are the serotonergic raphe neurons and brainstem astrocytes. For raphe neurons, recent elegant intersectional approaches have revealed remarkable molecular and functional diversity within the serotonergic system, and focused attention specifically on the Egr2-Pet1 subset of caudal raphe neurons as potential respiratory chemoreceptors. These particular neurons are directly CO_2_/H^+^ sensitive *in vitro*, an observation not yet verified *in vivo*, and inhibition of this subset of serotonergic cells blunts the HCVR. To date, TASK-1/TASK-3 channels are the only molecularly identified pH sensors in serotonergic raphe neurons, but genetic deletion of those TASK channels has no effect on the HCVR in mice. For astrocytes, there is good evidence that they are activated by CO_2_/H^+^ to mobilize intracellular Ca^2+^, but this has not been validated in conscious animals. Optogenetic activation of VMS astrocytes evokes ATP release and stimulates local RTN neurons and respiration via a P2Y receptor mechanism; conversely, inhibition of gliotransmitter release and ATP signaling in preBötC neurons blunts the HCVR, along with various other respiratory reflexes. It remains to be clarified whether there is a specific site for astrocytic modulation of CO_2_-dependent respiratory output, and the molecular specializations proposed to support CO_2_/H^+^ sensing by astrocytes have not yet been clearly linked to the HCVR. For LC and orexin neurons, which can modulate respiratory output and may indeed be CO_2_/H^+^ sensitive *in vitro*, there is much less direct evidence for the various criteria.

If this set of criteria can be fulfilled by one or more of these cell types and molecular sensors, then it will also be important to quantify their relative contributions and determine whether they function together in series, in parallel, or both. Our current working model holds that respiratory chemoreception and the HCVR is primarily subserved by a multicellular sensory apparatus. In particular, we see the RTN as both a direct CO_2_/H^+^ sensor and as a principal integrative center that transduces local environmental variations in CO_2_/H^+^ and neuromodulatory input from the other presumptive chemosensory cell groups for onward transmission to the respiratory rhythm and pattern generator circuits. These inputs modulate the excitability of RTN neurons, increasing their CO_2_/H^+^ sensitivity and input-output gain. To the extent that those other cell groups encode CO_2_/H^+^ *in vivo*, their inputs may confer a secondary CO_2_/H^+^ signal to RTN neurons while imparting their own chemosensitivity onto other elements of the respiratory control and output networks. Many predictions of this working model have not been directly tested, and those together with the chemoreceptor criteria we outlined here, can hopefully serve as a guide for future experiments. Regardless of whether any of these cell groups fulfill all the listed criteria for *bona fide* respiratory chemoreceptors, it is clear that they each provide important modulatory influences on downstream respiratory networks that enhance how changes in CO_2_ are ultimately translated into an effective homeostatic ventilatory response. Finally, it is also important to recognize that these cell groups could serve chemoreceptor functions for other non-respiratory effects of CO_2_ (arousal, anxiety, etc.).
